# Analysis of fractional order model on higher institution students’ anxiety towards mathematics with optimal control theory

**DOI:** 10.1038/s41598-023-33961-y

**Published:** 2023-04-27

**Authors:** Shewafera Wondimagegnhu Teklu

**Affiliations:** Department of Mathematics, Natural Science, Debre Berhan University, 445 Debre Berhan, Ethiopia

**Keywords:** Biological techniques, Computational biology and bioinformatics, Ecology, Environmental social sciences, Medical research, Mathematics and computing

## Abstract

Anxiety towards mathematics is the most common problem throughout nations in the world. In this study, we have mainly formulated and analyzed a Caputo fractional order mathematical model with optimal control strategies on higher institution students’ anxiety towards mathematics. The non-negativity and boundedness of the fractional order dynamical system solutions have been analysed. Both the anxiety-free and anxiety endemic equilibrium points of the Caputo fractional order model are found, and the local stability analysis of the anxiety-free and anxiety endemic equilibrium points are examined. Conditions for Caputo fractional order model backward bifurcation are analyzed whenever the anxiety effective reproduction number is less than one. We have shown the global asymptotic stability of the endemic equilibrium point. Moreover, we have carried out the optimal control strategy analysis of the fractional order model. Eventually, we have established the analytical results through numerical simulations to investigate the memory effect of the fractional order derivative approach, the behavior of the model solutions and the effects of parameters on the students anxiety towards mathematics in the community. Protection and treatment of anxiety infectious students have fundamental roles to minimize and possibly to eradicate mathematics anxiety from the higher institutions.

## Introduction

Learning ability and performance of school or higher institutions students can be influenced by different causes, including learning environment, instructional activities of teachers, attitude of students towards the discipline^1,2^. Skills abilities and knowledge of mathematics are fundamental for understanding other disciplines including engineering, sciences, social sciences and even the arts to school as well as higher institutions students^2,3^. Mathematics plays various roles in social sciences, natural sciences, engineering and thecnology, in the business world as well as in the success of communities throughout the world^2–4^.

Attitudes toward mathematics have been defned as beliefs that mathematics is important or useless or liking or disliking of mathematics, or a trend to engage in or quash mathematical activities^3^. School and higher institutions students attitudes including their belief, emotion, and behavior are most important and crucial for their accadamic achivements and optimize subject matter capability^3,5^. Instructional and social psychological environment are some of the students attitude attribute and possible factors affecting the students’ disliking or liking of mathematics and mathematics anxiety is closely related to a broad spectrum of cognitive, psychological, and behavioral problem^2,6^. Mathematics education is possibly a realistic approach to discover the relation between student achievement and attitudes towards mathematics^5^. Emotions towards students learning abilities and the teaching–learning environment conditions by the majority of school teachers and higher institutions lecturers can be improved by enhancement in teaching–learning environment conditions, using appropriate instructions in classroom, practicing best teaching skills, improve learning skills of students, giving training programs for teachers, improve attitude of the whole community^1,7^.

Students’ anxiety towards mathematics is defined as the negative perception of mathematics or a feeling of tension and apprehension that interferes with mathematics performance ability, the manipulation of numbers and solving of mathematical problems in a wide variety of ordinary life and academic situations. It has four dimensions such as anxiety of learning mathematics, anxiety of solving problem, anxiety of mathematics teacher, and anxiety on evaluation of mathematics knowledge and skills^8,9^. It exists in some adults and affects all aspects of teaching and learning mathematics^8,9^. Students who suffer from higher levels of mathematics anxiety typically develop negative attitudes and emotions toward mathematics that will consequently lead to a decreased level of achievement in mathematics^4^.

Studies of real world situations in different disciplines of social sciences, natural sciences, engineering and technology, businesses, and even the arts using mathematical modelling approaches have attracted the attention of various scholars because they provide a better understanding of their investigation, existence, stabilities, and controlling mechanisms^3,10,11^. In the past, a lot of mathematical models of real world situations are consist of a system of integer-order differential equations. Nevertheless, in the last few decades, the fractional-order differential equation approach has been applied in the modelling of real world situations since this approach provides a greater degree of accuracy than the integer order differential equation approach^10,11^. Recently, researchers’ interest to conduct a fractional order modelling research to investigate real world situations phenomenon in different disciplines increases because of a lot of properties which are not found in classical integer order modelling approach. The integer order modelling approach are local in nature, but, most of the fractional order modelling approach are non-local and assesses the memory effect which make itself more powerful than integer order modelling approach^10^. Due to this realistic property and their popularity to model the behavior of real systems in various disciplines, many scholars recently proposed a study of real world situations using fractional order modelling approach rather than integer order modelling approach^10,11^.

Different scholars throughout the world have been formulated and analyzed mathematical modeling on various real-world situations using deterministic modelling approach or stochastic modelling approach or fractional order modelling approach see references^12–14^. To construct and analyze our proposed study we have reviewed some scholar studies those are relevant and constructive to our study regarding to concepts, theories, methods and methodologies. Brahim et al.^15^ constructed a fractional order model to investigate the impact of harvesting on prey-predator interaction in the case of prey heard behavior. The researchers’ main purpose to use fractional order model is to model the memory effect measured by the order of the fractional derivative on the mutual interactions and to investigate the impact of inner competition among predators. Teklu and Terefe^3^ formulated and analyzed a susceptible, exposed, animosity-infected and treated (SEATS) compartments deterministic model on the dynamics of university students animosity towards mathematics with optimal control theory. The result concluded that applying prevention and treatment control measures simultaneously is the best strategy to minimize and possibly to eradicate the animosity-infection in the community. Mandal et al.^11^ formulated and analyzed a fractional-order epidemic model with fear effect of an infectious disease with treatment control. Also fractional backward and fractional Hopf bifurcation are also analyzed and shown a role the disease control parameter, level of fear and fractional order play in the stability of equilibriums and Hopf bifurcation.

Kumar et al.^16^ formulated and investigated a Caputo fractional order model on the alkali–silica reaction dynamics with six differential equations. The model analysis proved that the unique solution existence, the stability of the system using methods suitable for fractional order model approach, and illustrate the numerical simulation graphs to justify the analytical results using suitable numerical methods known as Adams- Bash forth–Moulton scheme. The study shows the significance of fractional order modelling approach in the study of chemical reactions. Erturk et al.^17^ formulated a fractional order modelling approach to investigate the motion of a beam on an internally bent nanowire. Results in the model analysis shows that the fractional responses approach the classical ones as the fractional order goes to unity and additionally the fractional Euler–Lagrange equation provides a flexible model possessing more information than the classical description. Viera-Martin et al.^18^ carried out a bibliographic analysis on artificial neural networks (ANNs) using fractional order modelling approach. They considered fractional order modelling approach to achieve the three objectives, such as systems stabilization, systems synchronization, and parameters training, using optimization algorithms. From the finding of the study they recommend that the Caputo fractional order modelling approach is the most utilized method for solving problems with ANNs because its initial values take the same form as the differential equations of integer-order approach. Kumar et al.^19^ proposed a delay Caputo fractional order model on the oncolytic virotherapy compositing viral lytic cycle and virusspecific cytotoxic T lymphocyte (CTL) response. From the analysis of the proposed model they concluded that a fractional order modelling approach has different behaviors as compared to the integer order modelling approach and the algorithm applied in the numerical analysis part smooth and reliable for delay fractional order approach. Din et al.^20^ proposed and analyzed a Caputo fractional order model on climate change. They carried out both the qualitative and numerical analysis of the proposed model and shows the result that the total spectrum lying between two integer values are achieved with more information about the complexity of the dynamics of the proposed fractional Climate Change-model. Based on the studies we have reviewed above we understand that the fractional order modelling approach could produce better solutions in the comparison of existing classical (integer order order) models, but the model analysis with fractional derivatives approach is more complicated than the classical integer order modelling approach. In this study the main and strong reason to use Caputo derivative in addition to its suitability for initial value problem, when talking about real problems, the Caputo derivative is highly useful since it allows traditional starting and boundary conditions be included in the derivation, and the derivative of a constant is zero that is not the case with the Riemann–Liouville fractional derivative^21^.

The aim of this research study is to formulate and analyze the new Caputo fractional order model with optimal control theory on the higher institutions students' anxiety towards mathematics transmission dynamics with prevention and control strategies. We are motivated with the above studies and high interest in finding the best strategies to control the higher institution students’ anxiety towards mathematics using fractional order modelling approach. To the best of our knowledge, there is no scholars who studied higher institutions students anxiety towards mathematics using fractional order modelling approach so that our proposed model is unique in the given research thematic area.The remaining part of this paper is organized as follows. In section two we discussed the basic mathematical preliminaries important for the study. In section three we formulate the integer order model where each parameter and human compartment was explained. In section four we re-formulated and analyzed the integer order model given in section three as the Caputo fractional-order model. In section five we carried out the optimal control analysis of the Caputo fractional-order model given in section four. In section six and section seven we carried out the numerical simulations of the integer order and fractional order models respectively. In section eight we have discussed.and concluded the whole process of the research study.

## Basic definitions of fractional calculus

In this section we recall some basic definitions of fractional calculus.

### Definition 1

The Caputo fractional order derivative with order $\theta$ for a function $h \in {C}^{n}$ is defined as^22,23^

1 $$^\mathrm{C}{D}_{t}^{\theta } \, h\left(t\right)=\frac{1}{\Gamma  \, \left(\mathrm{n}-\uptheta \right)}{\int }_{0}^{t}{h}^{n} \, \left(\xi \right) \, \frac{{\left(t-\xi \right)}^{n-\theta }}{t-\xi }d\xi , n-1<\theta \le n\in N.$$

Note:

2 $$^\mathrm{C}{D}_{t}^{\theta } \, h\left(t\right) \, \mathrm{ tends  \, to } \, \dot{h}\left(t\right) \, \mathrm{ as } \, \theta \to 1.$$

### Definition 2

The Caputo case fractional order integral with order $\alpha >0$ for a function $h \in {C}^{n}$ is defined as^22,23^

3 $$^\mathrm{C}{I}_{t}^{\theta }h\left(t\right)=\frac{1}{\Gamma \left(\uptheta \right)}{\int }_{0}^{t}h\left(\xi \right)\frac{{\left(t-\xi \right)}^{\theta }}{t-\xi }d\xi , 0<\theta <1, t>0.$$

### Definition 3

The Mittag–Leffler function in two parameters is defined by^22,23^

4 $${E}_{{\theta }_{1,}{\theta }_{2}}\left(t\right)=\sum_{k=1}^{\infty }\frac{{t}^{k}}{\Gamma \left({\uptheta }_{1}\mathrm{k}+{\theta }_{2}\right)},\mathrm{ where }{\theta }_{1}>0,{\theta }_{2}>0.$$

For ${\theta }_{2}=1$, the Mittag–Leffler function in one parameter is defined by^22^

5 $${E}_{{\theta }_{1},1}\left(t\right)=\sum_{k=1}^{\infty }\frac{{t}^{k}}{\Gamma \left({\uptheta }_{1}\mathrm{k}+1\right)}={E}_{{\theta }_{1}}\left(t\right).$$

### Definition 4

The constant point ${\delta }^{*}$ is an equilibrium point of the Caputo-fractional model, then

6 $$^\mathrm{C}{D}_{t}^{\theta }\delta \left(t\right)=h\left(t,\delta \left(t\right)\right), \theta \in \left[\mathrm{0,1}\right] \, \mathrm{ if  \, and  \, only \,  if } \, h\left(t,{\delta }^{*}\right)=0.$$

## Integer order model formulation

In this section, we briefly discuss the integer order model for the dynamics of higher institutions students’ anxiety towards mathematics which is in the case of Caputo case fractional order derivative. In this study, to analyze the higher institutions students anxiety towards mathematics, we divide the total number of higher institution students denoted by $N(t)$ into six distinct groups such as students who are susceptible to anxiety towards mathematics denoted by ${S}_{h} \left(t\right)$, students who are protected from anxiety towards mathematics denoted by ${P}_{h} \left(t\right),$ students exposed to anxiety towards mathematics denoted by ${E}_{h}(t)$, students who have anxiety towards mathematics denoted by ${A}_{h}(t)$, students who have a permanent anxiety towards mathematics denoted by ${Q}_{h}(t)$, and students who are recovered from anxiety towards mathematics denoted by ${R}_{h}(t)$ such that

7 $$N\left(t\right)= {S}_{h}\left(t\right)+{ P}_{h} \left(t\right)+{E}_{h} \left(t\right)+{A}_{h} \left(t\right)+{Q}_{h}\left(t\right)+{R}_{h}\left(t\right).$$

In this mathematical model formulation, since anxiety towards mathematics is not density-dependent students susceptible to anxiety towards mathematics acquire anxiety at the standard incidence rate given by

8 $${\uplambda }_{\mathrm{n}}\left(\mathrm{t}\right)=\frac{\upbeta }{N}{(A}_{h}\left(t\right)+{\varphi Q}_{h}\left(t\right)).$$

Basic assumptions to develop the students anxiety towards mathematics transmission dynamics model: $\varepsilon$ is portion of the recruited students who are entered to protected group, the susceptible group is increasing by the new recruitment portion $(1-\varepsilon )$, students from protected group in which those higher institution students who are protected but who lost protection by the rate $(1-\kappa )\rho$, $\upkappa$ is efficiency of protection and from recovered group who lose their temporary recovery by the rate $\omega$, the student population is homogeneous in every group, estudents in each group are subject to natural death rate $\mu$, student population is variable, anxiety can naturally recovered, students can recovered from anxiety after treatment measure and some students will have permanent anxiety towards mathematics. The student population is homogeneous and variable.

**Figure 1:**
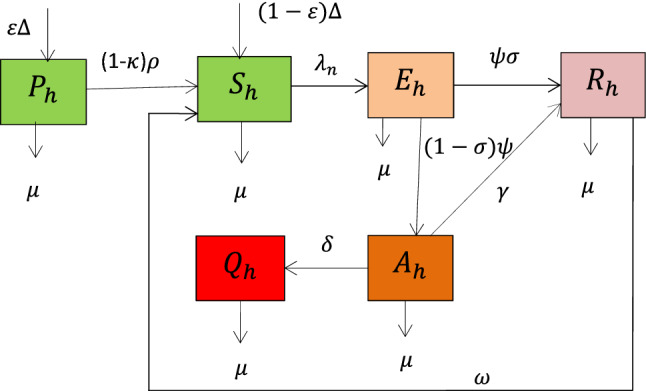
The flow diagram of transmission dynamics of higher institutions students anxiety towards mathematics where ${\lambda }_{n}$ is given in (Eq. 8).

Using parameters in Table 1, variables in Table 2, and assumptions described above the transmission flow diagram of higher institutionse students mathematics anxiety towards mathematics is given in Fig. 1

**Table 1:** Interpretation of the model parameters.

Parameter interpretation	
$\mu$	Human natural mortality rate
$\Delta$	Human recruitment rate
$\psi$	Probability of exposed students to be recovered
$\delta$	Progression rate to permanent anxiety infected group
$\gamma$	Treatment rate of anxiety infected
$\rho$	Anxiety protection lose rate
$\varepsilon$	Protection rate
$\beta$	Anxiety transmission rate
$\upomega$	Anxiety recovery lose rate
$\sigma$	Rate of exposed students who are leaving out of the group
$\kappa$	Effeciency of protection mechanism

**Table 2:** Definitions of state variables.

Variable definition	
${S}_{h}$ Anxiety towards mathematics susceptible students	
${P}_{h}$	Anxiety towards mathematics protected students
${E}_{h}$	Anxiety towards mathematics exposed students
${A}_{h}$	Students who have anxiety towards mathematics
${Q}_{h}$	Students who have permanent anxiety towards mathematics
${R}_{h}$	Students recovered from anxiety towards mathematics

From Fig. 1 the nonlinear ordinary differential equations governed by the model assumptions are described as follows:

9 $$ \begin{aligned} & \frac{d{S}_{h}}{dt}=(1-\varepsilon )\Delta +\omega {R}_{h}+\uprho (1-\upkappa ){P}_{h}-\left({\lambda }_{n}+\mu \right){S}_{h},\\ & \frac{d{P}_{h}}{dt}=\varepsilon\Delta -\left(\mu +(1-\kappa \right)\rho ){P}_{h},\\ &\frac{d{E}_{h}}{dt}={\lambda }_{n}{\mathrm{S}}_{h}-\left(\mu +\psi \right){E}_{h},\\ & \frac{{\mathrm{dA}}_{h}}{\mathrm{dt}}=\left(1-\upsigma \right)\uppsi {\mathrm{E}}_{h}-\left(\upmu +\updelta +\upgamma \right){A}_{h},\\ &\frac{d{Q}_{h}}{dt}=\delta {A}_{h}-\mu {Q}_{h},\\ &   \frac{d{R}_{h}}{dt}=\gamma {A}_{h}+\sigma \psi {E}_{h}-(\mu +\omega ){R}_{h}, \end{aligned}$$

with initial conditions

10 $${S}_{h}\left(0\right)>0, { P}_{h}\left(0\right)\ge 0, {E}_{h}\left(0\right)\ge 0, {A}_{h}\left(0\right)\ge 0, {Q}_{h}\left(0\right)\ge 0,\mathrm{ and }{R}_{h}\left(0\right)\ge 0.$$

The sum of all the differential equations in (Eq. 2) is $\frac{dN}{dt}=\Delta -dN.$

## Model derivation in Caputo fractional order derivative approach

In this section, we re-formulate the higher institutions students anxiety towards mathematics model (Eq. 9) using a Caputo fractional order derivative approach in order to observe the memory effects and gain more insights about the dynamics of higher institutions students anxiety towards mathematics. System (9) in terms of integral form is followed by substituting the value of kernel as a power-law correlation function. After applying the Caputo fractional derivative of $(\theta -1),$ we have got

11 $$ \begin{aligned} & ^\mathrm{C}{D}_{t}^{\theta -1}\left[\frac{d{S}_{h}}{dt}\right]=^\mathrm{C}{D}_{t}^{\theta -1}{I}_{t}^{\theta -1}[(1-\varepsilon )\Delta +\omega {R}_{h}+(1-\upkappa )\uprho {P}_{h}-\left({\lambda }_{n}+\mu \right){S}_{h}],\\ & ^\mathrm{C}{D}_{t}^{\theta -1}\left[\frac{d{P}_{h}}{dt}\right]=^\mathrm{C}{D}_{t}^{\theta -1}{I}_{t}^{\theta -1}[\varepsilon\Delta -\left(\mu +1-\upkappa )\rho \right){P}_{h}], \\ & ^\mathrm{C}{D}_{t}^{\theta -1}\left[\frac{d{E}_{h}}{dt}\right]=^\mathrm{C}{D}_{t}^{\theta -1}{I}_{t}^{\theta -1}[{\lambda }_{n}{\mathrm{S}}_{h}-\left(\mu +\psi \right){E}_{h}],\\ & ^\mathrm{C}{D}_{t}^{\theta -1}\left[\frac{d{A}_{h}}{dt}\right]=^\mathrm{C}{D}_{t}^{\theta -1}{I}_{t}^{\theta -1}[(1-\sigma )\psi {E}_{h}-\left(\mu +\delta +\gamma \right){A}_{h}], \\ & ^\mathrm{C}{D}_{t}^{\theta -1}\left[\frac{d{Q}_{h}}{dt}\right]=^\mathrm{C}{D}_{t}^{\theta -1}{I}_{t}^{\theta -1}[\delta {A}_{h}-\mu {Q}_{h}],\\ & ^\mathrm{C}{D}_{t}^{\theta -1}\left[\frac{d{R}_{h}}{dt}\right]=^\mathrm{C}{D}_{t}^{\theta -1}{I}_{t}^{\theta -1}[\gamma {A}_{h}+\psi \sigma {E}_{h}-\left(\mu +\omega \right){R}_{h}]. \end{aligned}$$

Both the left and right side of Eq. (11) are the inverse operators, we have the higher institutions students anxiety towards mathematics daynamical system (Eq. 9) in the Caputo fractional order operator form

12 $$\begin{aligned}& ^\mathrm{C}{D}_{t}^{\theta }{S}_{h}= (1-{\varepsilon }^{\theta }){\Delta }^{\theta }+{\omega }^{\theta }{R}_{h}+(1-{\upkappa }^{\theta }){\uprho }^{\theta }{P}_{h}-\left( {\lambda }_{n}+{\mu }^{\theta }\right){S}_{h}, \\ &^\mathrm{C}{D}_{t}^{\theta }{P}_{h}={\varepsilon }^{\theta }{\Delta }^{\theta }-\left({\mu }^{\theta }+(1-{\upkappa }^{\theta }){\rho }^{\theta }\right){P}_{h},\\ & ^\mathrm{C}{D}_{t}^{\theta }{E}_{h}={\lambda }_{n}{\mathrm{S}}_{h}-\left({\mu }^{\theta }+{\psi }^{\theta }\right){E}_{h}, \\ & ^\mathrm{C}{D}_{t}^{\theta }{A}_{h}=(1-{\sigma }^{\theta }){\psi }^{\theta }{E}_{h}-\left({\mu }^{\theta }+{\delta }^{\theta }+{\gamma }^{\theta }\right){A}_{h},\\ &^\mathrm{C}{D}_{t}^{\theta }{Q}_{h}={\delta }^{\theta }{A}_{h}-{\mu }^{\theta }{Q}_{h},\\ &    ^\mathrm{C}{D}_{t}^{\theta }{R}_{h}=\gamma {A}_{h}+{\psi }^{\theta }{\sigma }^{\theta }{E}_{h}-\left({\mu }^{\theta }+{\omega }^{\theta }\right){R}_{h}. \end{aligned}$$

The initial conditions for the fractional order derivatives (Eq. 8) are given as:

13 $$\begin{aligned}& {S}_{h}\left(0\right)\ge 0,{P}_{h}\left(0\right)\ge 0,{E}_{h}(0)\ge 0,\\ &{A}_{h}\left(0\right)\ge 0,{Q}_{h}\left(0\right)\ge 0,{R}_{h}(0)\ge 0.\end{aligned}$$

### Basic properties of the Cauto fractional model (Eq. 12)

In the study, since we are dealing with the number of higher institutions students which cannot be negative, we have to show there is non-negative solution in the given region to the fractional order model (Eq. 12). The constructed model is mathematically analysed by proving different theorems and algebraic computation dealing with different quantitative and qualitative attributes.

#### Theorem 1 (Positivity of the model solutions)

*Each solution to the fractional order model * (Eq. 12) *with initial conditions* ${S}_{h}(0)\ge 0,$ ${P}_{h}(0)\ge 0,$ ${E}_{h}(0)\ge 0,{A}_{h}(0)\ge 0,$ ${Q}_{h}(0)\ge 0,$ ${R}_{h}(0)\ge 0$ (Eq. 13) *is positive*.

#### Proof

Using similar approach stated in reference^24^, we assume by contradiction that the first equation in fractional dynamic model (Eq. 12) is not true.

Let ${t}_{*}=min\left\{t: {S}_{h}\left(t\right){P}_{h}\left(t\right){E}_{h}\left(t\right){A}_{h}\left(t\right){Q}_{h}\left(t\right){R}_{h}\left(t\right)=0\right\}$. Now if ${S}_{h}\left({t}_{*}\right)=0,$ it then ${P}_{h}\left(t\right)\ge 0$,${E}_{h}\left(t\right)\ge 0$, ${A}_{h}\left(t\right)\ge 0$, ${Q}_{h}\left(t\right)\ge 0$, and ${R}_{h}\left(t\right)\ge 0$ for all $0\le t\le {t}_{*}$.

Let us consider the following expression and assume it exists,

$${A}_{1}=\underset{0\le t\le {t}_{*}}{\mathrm{min}}\left\{\frac{1}{{S}_{h}}( \left(1-{\varepsilon }^{\theta }\right){\Delta }^{\theta }+{\omega }^{\theta }{R}_{h}+\left(1-{\upkappa }^{\theta }\right){\uprho }^{\theta }{P}_{h})-\left( {\lambda }_{n}+{\mu }^{\theta }\right)\right\}.$$

It follows that

^C^${D}_{t}^{\theta }{S}_{h}(t)$-${A}_{1}{S}_{h}\left(t\right)\ge 0.$ Applying Laplace transform we have determine that

$${s}^{\theta }\stackrel{\smile}{{S}_{h}}\left(s\right)-{s}^{\theta -1}{S}_{h}\left(0\right)-{A}_{1}\stackrel{\smile}{{S}_{h}}\left(s\right)\ge 0.$$

So that $\stackrel{\smile}{{S}_{h}}\left(s\right)\ge {S}_{h}\left(0\right)\frac{{s}^{\theta -1}}{{s}^{\theta }-{A}_{1}}=\frac{{S}_{h}\left(0\right)}{s}{\left(1-\frac{{A}_{1}}{{s}^{\theta }}\right)}^{-1}={S}_{h}\left(0\right)\sum_{k=0}^{\infty }\frac{{{A}_{1}}^{k}}{{s}^{k\theta +1}}$.Using the inverse Laplace transform on this last expression and expressed as a Mit-tag–Leffler function of one parameter, we have determine that ${S}_{h}\left(t\right)\ge {S}_{h}\left(0\right)\sum_{k=0}^{\infty }\frac{{\left({A}_{1}{t}^{\theta }\right)}^{k}}{\Gamma \left(k\theta +1\right)}={S}_{h}\left(0\right){E}_{\theta }\left({A}_{1}{t}^{\theta }\right)$. Thus, the positivity of the solution ${S}_{h}$ is represented by ${S}_{h}\left(t\right)\ge {S}_{h}\left(0\right){E}_{\theta }\left({A}_{1}{t}^{\theta }\right)\ge 0$ which contradicts our assumption ${S}_{h}\left({t}_{*}\right)=0$. In a similar manner one can show that for each remaing state variable and hence each solution ${S}_{h}\left(t\right), {P}_{h}\left(t\right),{E}_{h}\left(t\right), {A}_{h}\left(t\right), {Q}_{h}\left(t\right)$, and ${R}_{h}\left(t\right)$ of the fractional order model (Eq. 12) is positive. Therefore, since we have shown the solution is bounded and positive, we can reasonably say that the model is both biologically and mathematically meaningful.

#### Theorem 2 (Boundedness of the fractional order model solutions)

* The fractional order model* (Eq. 12) *solutions are bounded in the region* $$\Omega =\left\{\begin{array}{c}\left({S}_{h},{P}_{h},{E}_{h},{A}_{h},{Q}_{h},{R}_{h}\right)\in {\mathbb{R}}_{+}^{6},N(t)\le \frac{\Delta }{\mu }\end{array}\right\}$$.

#### Proof

By adding all the fractional order derivatives in Eq. (12) we have determined as

$$ \begin{aligned}& ^{\rm C}{D}_{t}^{\theta }N\left(t\right)= \, ^{\rm C}{D}_{t}^{\theta }{S}_{h}+ ^{\rm C}{D}_{t}^{\theta }{P}_{h}+ ^{\rm C} {D}_{t}^{\theta }{Q}_{h}+^{\rm C} {D}_{t}^{\theta }{R}_{h}\le\Delta -\mu N(t) \\  &  \Rightarrow {D}_{t}^{\theta }N\left(t\right)\le {\Delta }^{\theta }- {\mu }^{\theta }N\left(t\right). \end{aligned}$$

Applying Laplace transformation on both sides gives $\mathcal{L}\left({D}_{t}^{\theta }N\left(t\right)\right)\le \frac{{\Delta }^{\theta }}{S}-{\mu }^{\theta }\mathcal{L}\left(N\left(t\right)\right)$. Simplifying the result to determine the following inequality $\mathcal{L}\left(N\left(t\right)\right)\le \frac{\Delta {\mathrm{S}}^{-1}}{{S}^{\theta }+{\mu }^{\theta }}+\frac{{\mathrm{S}}^{\theta -1}N(0)}{{S}^{\theta }+{\mu }^{\theta }}$.And solving using the definition of inverse Laplace transformation and using definition (Eq. 5) we have determined the expression given by $N\left(t\right)\le N\left(0\right){E}_{\theta }\left(-{\mu }^{\theta }{t}^{\theta }\right)+\frac{{\Delta }^{\theta }}{{\mu }^{\theta }}\left(1-{E}_{\theta }\left(-{\mu }^{\theta }{t}^{\theta }\right)\right)$. Using definition (Eq. 5) if $N\left(0\right)\le \frac{{\Delta }^{\theta }}{{\mu }^{\theta }}$, then $0<N\left(t\right)\le \frac{{\Delta }^{\theta }}{{\mu }^{\theta }}$ for each $t\ge 0.$ Therefore, the total number of students population $N\left(t\right)$ is bounded and hence the proposed fractional order model (Eq. 12) is both mathematically and biologically meaningful.

### Qualitative analysis of the fractional order model (Eq. 12)

#### Anxiety-free equilibrium point of the model (Eq. 12)

For the higher institutions students anxiety-free equilibrium point of the fractional order model (Eq. 12), we have calculated from ^C^${D}_{t}^{\theta }{S}_{h}\left(t\right)=$ ^C^ ${D}_{t}^{\theta }{P}_{h}\left(t\right)$ = ^C^${D}_{t}^{\theta }{E}_{h}\left(t\right)$ = ^C^${D}_{t}^{\theta }{A}_{h}\left(t\right)$= ^C^${D}_{t}^{\theta }{Q}_{h}\left(t\right)$= ^C^${D}_{t}^{\theta }{R}_{h}\left(t\right)$=0. Then after some steps of computations the higher institutions students anxiety-free equilibrium point of the fractional model (Eq. 12) is given by

$${E}_{A}^{0}=\left({S}_{h}^{0},{P}_{h}^{0},{E}_{h}^{0},{A}_{h}^{0},{Q}_{h}^{0}{,R}_{h}^{0}\right)=\left(\frac{{\Delta }^{\theta }(1-{ \varepsilon }^{\vartheta }){\mu }^{\theta }+(1-{\upkappa }^{\theta }){\rho }^{\theta })}{{\mu }^{\theta }\left({\mu }^{\theta }+\left(1-{\upkappa }^{\theta }\right){\rho }^{\theta }\right)} ,\frac{{\varepsilon }^{\theta }{\Delta }^{\theta }}{{\mu }^{\theta }+\left(1-{\upkappa }^{\theta }\right){\rho }^{\theta }},0,\mathrm{0,0},0 \right).$$

#### Effective reproduction number of the model (Eq. 12)

In this manuscript the next generation matrix operator approach by Van den Driesch and Warmouth as in^25^ is used to derive the effective reproduction number ${\mathcal{R}}_{eff}^{\theta }$ for the dynamics of the higher institutions students anxiety towards mathematics. The higher institutions students anxiety towards mathematics model (Eq. 12) effective reproduction number denoted by ${\mathcal{R}}_{eff}^{\theta }$ measures the average number of the higher institutions anxiety infected students generated by one anxiety infected student in a considered community of students when some controlling strategies are in place, like anxiety protection, or/and anxiety treatment. The students anxiety infection effective reproduction number denoted by the symbol ${\mathcal{R}}_{eff}^{\theta }$ is the dominant (largest) eigenvalue (spectral radius) of the matrix $F{V}^{-1}=\left[\frac{\partial {\mathcal{F}}_{i}({E}_{A}^{0})}{\partial {x}_{j}}\right]{\left[\frac{\partial {\nu }_{i}({E}_{A}^{0})}{\partial {x}_{j}}\right]}^{-1}$ where ${\mathcal{F}}_{i}$ is the rate at which newly anxiety infected students appear in compartment $i$, ${\nu }_{i}$ is the transfer of students who have anxiety towards mathematics from existing compartment $i$ to another compartment and ${E}_{A}^{0}$ is the students anxiety-free equilibrium point. Let $x={({E}_{h},{A}_{h},{Q}_{h})}^{T}$, then we have $\frac{dx}{dt}=F-V$ and after a detailed computations, we have got the transmission matrix as.

$$F=\left[\begin{array}{ccc}0& \frac{{\beta }^{\theta }{S}_{h}^{0}}{{S}_{h}^{0}+{P}_{h}^{0}}& \frac{{\beta }^{\theta }{\varphi }^{\theta }{S}_{h}^{0}}{{S}_{h}^{0}+{P}_{h}^{0}}\\ 0& 0& 0\\ 0& 0& 0\end{array}\right],$$

and the transition matrix as

$$V=\left[\begin{array}{c}{\mu }^{\theta }+{\uppsi }^{\theta }\\ -\left(1-{\upsigma }^{\theta }\right){\uppsi }^{\theta }\\ 0\end{array} \begin{array}{cc}0& 0\\ {\mu }^{\theta }+{\delta }^{\theta }+{\gamma }^{\theta }& 0\\ -{\delta }^{\theta }& {\mu }^{\theta }\end{array}\right].$$

The next generation matrix is of the form

$$F{V}^{-1}=\left[\begin{array}{ccc}\frac{{\beta }^{\theta }{\alpha }^{\theta }{S}_{h}^{0}}{{ D}_{1}({S}_{h}^{0}+{P}_{h}^{0})}+\frac{{\beta }^{\theta }{\mathrm{\varphi }}^{\theta }{\alpha }^{\theta } {\delta }^{\theta } {S}_{h}^{0}}{{\mu }^{\theta }{ D}_{1}({S}_{h}^{0}+{P}_{h}^{0})}& \frac{{\beta }^{\theta }{S}_{h}^{0}}{({\mu }^{\theta }+{\delta }^{\theta }+{\gamma }^{\theta })({S}_{h}^{0}+{P}_{h}^{0})}+\frac{{\beta }^{\theta }{\mathrm{\varphi }}^{\theta }{\delta }^{\theta }{S}_{h}^{0}}{{\mu }^{\theta }({\mu }^{\theta }+{\delta }^{\theta }+{\gamma }^{\theta })({S}^{0}+{P}_{h}^{0})}& \frac{{\beta }^{\theta }{\mathrm{\varphi }}^{\theta }{S}_{h}^{0}}{{\mu }^{\theta }({S}_{h}^{0}+{P}_{h}^{0})}\\ 0& 0& 0\\ 0& 0& 0\end{array}\right].$$

where $${S}_{h}^{0}=\frac{{\Delta }^{\theta }(1-{ \varepsilon }^{\vartheta }){\mu }^{\theta }+(1-{\upkappa }^{\theta }){\rho }^{\theta })}{{\mu }^{\theta }({\mu }^{\theta }+(1-{\upkappa }^{\theta }){\rho }^{\theta })} , {P}^{0}=\frac{{\varepsilon }^{\theta }{\Delta }^{\theta }}{{\mu }^{\theta }+(1-{\upkappa }^{\theta }){\rho }^{\theta }}$$ and $${D}_{1}=\left({\mu }^{\theta }+{\uppsi }^{\theta }\right)\left({\mu }^{\theta }+{\delta }^{\theta }+{\gamma }^{\theta }\right).$$

Then the spectral radius of the matrix $F{V}^{-1}$ which is called the higher institutions anxiety infected students model (Eq. 12) effective reproduction number given by

$${\mathcal{R}}_{eff}^{\theta }=\frac{{\beta }^{\theta }(1-{\sigma }^{\theta }){\uppsi }^{\theta }{S}_{h}^{0}}{{ D}_{1}({S}_{h}^{0}+{P}_{h}^{0})}+\frac{{\beta }^{\theta }{\mathrm{\varphi }}^{\theta }(1-{\sigma }^{\theta }){\uppsi }^{\theta }{\delta }^{\theta }{S}_{h}^{0}}{{\mu }^{\theta }{ D}_{1}({S}_{h}^{0}+{P}_{h}^{0})}=\frac{{\beta }^{\theta }(1-{\sigma }^{\theta }){\uppsi }^{\theta }\left(\left(1-{\varepsilon }^{\theta }\right){\mu }^{\theta }+(1-{\upkappa }^{\theta }){\rho }^{\theta }\right)[{\mu }^{\theta }+{\varphi }^{\theta }{\delta }^{\theta }]}{{\mu }^{\theta }\left({\mu }^{\theta }+\uppsi \right)\left({\mu }^{\theta }+{\delta }^{\theta }+{\gamma }^{\theta }\right)\left({\mu }^{\theta }+(1-{\upkappa }^{\theta }){\rho }^{\theta }\right)}$$

#### Local stability of anxiety-free equilibrium point

##### Theorem 3

*Given a fractional-order system of differential equation* ${D}_{0}^{\theta }y\left(t\right)=f\left(y\right),$ $0<\theta \le 1.$

Let ${y}_{0}$ be an equilibrium point of the dynamical system, and let $B=D(f\left({y}_{0}\right))$ be the Jacobian matrix of $f$ evaluated at ${y}_{0}$. Then ${y}_{0}$ is locally asymptotically stable if and only if $\left|\mathrm{arg}({\lambda }_{i})\right|>\frac{\theta \pi }{2}$, for each eigenvalue of the matrix $B$^22^.

Note: The proof of Theorem 2 illustrates that in case $0<\theta <1$, the stability region of the fractional-order model (Eq. 12) increases as compared to the integer order model.

##### Theorem 4

*The anxiety-free equilibrium point* ${E}_{A}^{0}$ *of the fractional order model* (Eq. 12) *is locally asymptotically stable if* ${\mathcal{R}}_{eff}^{\theta }<1$ *otherwise it is unstable*.

##### Proof

The local stability of the anxiety-free equilibrium point of the model (Eq. 12) at the anxiety-free equilibrium point ${E}_{A}^{0}=({S}_{h}^{0},{P}_{h}^{0},{E}_{h}^{0},{A}_{h}^{0},{Q}_{h}^{0}{,R}_{h}^{0}$) = $\left(\frac{{\Delta }^{\theta }(1-{ \varepsilon }^{\vartheta }){\mu }^{\theta }+(1-{\upkappa }^{\theta }){\rho }^{\theta })}{{\mu }^{\theta }\left({\mu }^{\theta }+\left(1-{\upkappa }^{\theta }\right){\rho }^{\theta }\right)} ,\frac{{\varepsilon }^{\theta }{\Delta }^{\theta }}{{\mu }^{\theta }+\left(1-{\upkappa }^{\theta }\right){\rho }^{\theta }},0,\mathrm{0,0},0 \right)$has been studied using the criteria stated in Theorem 3.

The Jacobian matrix of the dynamical system given in (Eq. 12) at the anxiety-free equilibrium point is given by

$$J\left({E}_{A}^{0}\right)$$=$$\left(\begin{array}{cccccc}-{\upmu }^{\theta }& {\uprho }^{\theta }& 0& -\frac{{\beta }^{\theta }{S}_{h}^{0}}{{S}^{0}+{P}_{h}^{0}}& -\frac{{\beta }^{\theta }{\mathrm{\varphi }}^{\theta }{S}_{h}^{0}}{{S}^{0}+{P}_{h}^{0}}& \omega \\ 0& -\left({\upmu }^{\theta }+(1-{\upkappa }^{\theta }){\rho }^{\theta }\right)& 0& 0& 0& 0\\ 0& 0& -\left({\mu }^{\theta }+{\uppsi }^{\theta }\right)& \frac{{\beta }^{\theta }{S}_{h}^{0}}{{S}_{h}^{0}+{P}_{h}^{0}}& \frac{{\beta }^{\theta }{\mathrm{\varphi }}^{\theta }{S}_{h}^{0}}{{S}_{h}^{0}+{P}_{h}^{0}}& 0\\ 0& 0& \left(1-{\upsigma }^{\theta }\right){\uppsi }^{\theta }& -\left({\mu }^{\theta }+{\delta }^{\theta }+{\gamma }^{\theta }\right)& 0& 0\\ 0& 0& 0& {\delta }^{\theta }& -{\mu }^{\theta }& 0\\ 0& 0& {\upsigma }^{\theta }{\uppsi }^{\theta }& {\gamma }^{\theta }& 0& -({\mu }^{\theta }+{\omega }^{\theta })\end{array}\right)$$.

The computations for eigenvalues of the Jacobian matrix $J\left({E}_{A}^{0}\right)$ gives

$${\lambda }_{1}=-\left({\upmu }^{\theta }+\left(1-{\upkappa }^{\theta }\right){\rho }^{\theta }\right), {\lambda }_{2}=-\left({\mu }^{\theta }+{\omega }^{\theta }\right), {\lambda }_{3}=-{\upmu }^{\theta },$$

and

14 $${a}_{3}{\lambda }^{3}+{a}_{2}{\lambda }^{2}+{a}_{1}\lambda +{a}_{0}=0$$

where

$$\begin{aligned} & {a}_{3}=1, {a}_{2}=\left({\mu }^{\theta }+{\uppsi }^{\theta }\right)+{\mu }^{\theta }\left({\mu }^{\theta }+{\delta }^{\theta }+{\gamma }^{\theta }\right)\\ & {a}_{1}={\mu }^{\theta }\left({\mu }^{\theta }+{\uppsi }^{\theta }\right)+ {\mu }^{\theta }\left({\mu }^{\theta }+{\delta }^{\theta }+{\gamma }^{\theta }\right)+\frac{\beta \mathrm{\varphi }\delta {S}_{h}^{0}}{{S}_{h}^{0}+{P}_{h}^{0}}+{\mu }^{\theta }\left({\mu }^{\theta }+{\delta }^{\theta }+{\gamma }^{\theta }\right)\left({\mu }^{\theta }+{\uppsi }^{\theta }\right)[1-{\mathcal{R}}_{eff}^{\theta }],\\ & {\rm and}\\ & {a}_{0}= {\mu }^{\theta }\left({\mu }^{\theta }+{\delta }^{\theta }+{\gamma }^{\theta }\right)\left({\mu }^{\theta }+{\uppsi }^{\theta }\right)[1-{\mathcal{R}}_{eff}^{\theta }].\end{aligned} $$

We can justify that ${a}_{0}$, ${a}_{1}$,${a}_{2}$ and ${a}_{3}$ are all positive implies all the three eigenvalues of Eq. (14) have negative real parts if ${\mathcal{R}}_{eff}^{\theta }<1.$ Then since all the eigenvalues of the Jacobian matrix $J\left({E}_{A}^{0}\right)$ have negative values using the criteria stated in Theorem 3 above $\left|\mathrm{arg}({\lambda }_{i})\right|>\frac{\theta \pi }{2}$ for $0<\theta \le 1$ and each $i=\mathrm{1,2},\mathrm{3,4},5,$ and 6. Thus, the anxiety-free equilibrium point of the model (Eq. 12) is locally asymptotically stable whenever ${\mathcal{R}}_{eff}^{\theta }<1.$

#### Existence of anxiety endemic equilibrium point (s) and bifurcation analysis

The anxiety endemic equilibrium point occurs when the anxiety exist in the higher institution student population. Anxiety endemic equilibrium point of the Caputo fractional order derivative model (Eq. 12) is obtained by making right hand side of all equations of system (Eq. 12) equal to zero. Let ${E}_{A}^{*}=({S}_{h}^{*},{P}_{h}^{*},{E}_{h}^{*},{A}_{h}^{*},{Q}_{h}^{*},{R}_{h}^{*})$ be the anxiety persistence (endemic) equilibrium point of model (Eq. 12) and ${\uplambda }_{n}^{*}\left(\mathrm{t}\right)=\frac{{\upbeta }^{\theta }}{{N}^{*}}({\mathrm{A}}_{h}^{*}\left(\mathrm{t}\right)+{\mathrm{\varphi }}^{\theta }{\mathrm{Q}}_{h}^{*}\left(\mathrm{t}\right))$ be the students anxiety standared incidence rate (“force of anxiety infection”) at the anxiety endemic equilibrium point. To find equilibrium point(s) for which anxiety infection is endemic in the population, the model equations given in Eq. (12) are solved in terms of ${\uplambda }_{n}^{*}\left(\mathrm{t}\right)=\frac{{\upbeta }^{\theta }}{{N}^{*}}({\mathrm{A}}_{h}^{*}\left(\mathrm{t}\right)+{\mathrm{\varphi }}^{\theta }{\mathrm{Q}}_{h}^{*}\left(\mathrm{t}\right))$ at an endemic equilibrium point. Now setting the right-hand sides of the equations of the model (Eq. 12) to zero as follows

15 $$ \begin{aligned} & ^{\rm C}{D}_{t}^{\theta }{S}_{h}^{*}=(1-{\varepsilon }^{\theta }){\Delta }^{\theta }+{\omega }^{\theta }{R}_{h}^{*}+(1-{\upkappa }^{\theta }){\uprho }^{\theta }{P}_{h}^{*}-\left({\uplambda }_{n}^{*}+{\mu }^{\theta }\right){S}_{h}^{*}=0,\\ &^{\rm C}{D}_{t}^{\theta }{P}_{h}^{*}{=\varepsilon }^{\theta }{\Delta }^{\theta }-\left({\mu }^{\theta }+(1-{\upkappa }^{\theta }){\rho }^{\theta }\right){P}_{h}^{*}=0,\\ & ^{\rm C}{D}_{t}^{\theta }{E}_{h}^{*}={\uplambda }_{n}^{*}{S}_{h}^{*}-\left({\mu }^{\theta }+{\uppsi }^{\theta }\right){E}_{h}^{*}=0,\\ & {^{\rm C}D}_{t}^{\theta }{A}_{h}^{*}=\left(1-{\upsigma }^{\theta }\right){\uppsi }^{\theta }{E}_{h}^{*}-\left({\mu }^{\theta }+{\delta }^{\theta }+{\gamma }^{\theta }\right){A}_{h}^{*}=0,\\ & ^{\rm C}{D}_{t}^{\theta }{Q}_{h}={\delta }^{\theta }{A}_{h}^{*}-{\mu }^{\theta }{Q}_{h}^{*}=0,\\ &^{\rm C}{D}_{t}^{\theta }{R}_{h}^{*}{=\gamma }^{\theta }{A}_{h}^{*}+{\sigma }^{\theta }{\uppsi }^{\theta }{E}_{h}^{*}-\left({\mu }^{\theta }+{\omega }^{\theta }\right){R}_{h}^{*}=0 .\end{aligned}  $$

After solving Eq. (15) we have determined the anxiety endemic point (s) as

16 $$ \begin{aligned}&{S}_{h}^{*}=\frac{{m}_{8}}{{m}_{9}{\uplambda }_{n}^{*}+{m}_{10}}, {P}_{h}^{*}=\frac{{\varepsilon }^{\theta }{\Delta }^{\theta }}{{m}_{3}} , {E}_{h}^{*}=\frac{{m}_{8}{\uplambda }_{n}^{*}}{{m}_{11}{\uplambda }_{n}^{*}+{m}_{12}}, {A}_{h}^{*}=\frac{{m}_{5}{m}_{8}{\uplambda }_{n}^{*}}{{m}_{13}{\uplambda }_{n}^{*}+{m}_{14}}, \\ & {Q}_{h}^{*}=\frac{{m}_{5}{m}_{8}\delta {\uplambda }_{n}^{*}}{{m}_{15}{\uplambda }_{n}^{*}+{m}_{16}}, {Q}_{h}^{*}=\frac{{{m}_{5}\gamma m}_{8}{\uplambda }_{n}^{*}+{{m}_{6}\sigma \mathrm{\psi \lambda }m}_{8}{\uplambda }_{n}^{*}}{{m}_{4}{m}_{6}{m}_{7}({m}_{9}{\uplambda }_{n}^{*}+{m}_{10})}. \end{aligned}$$

where

17 $$ \begin{aligned} & {m}_{1}=\left(1-{\varepsilon }^{\theta }\right){\Delta }^{\theta }, {m}_{2}=\left(1-{\upkappa }^{\theta }\right){\uprho }^{\theta }, {m}_{3}=\left({\mu }^{\theta }+(1-{\upkappa }^{\theta }){\rho }^{\theta }\right), {m}_{4}=\left({\mu }^{\theta }+{\uppsi }^{\theta }\right), {m}_{5}=\left(1-{\upsigma }^{\theta }\right){\uppsi }^{\theta },\\ & {m}_{6}=\left({\mu }^{\theta }+{\delta }^{\theta }+{\gamma }^{\theta }\right),  {m}_{7}=\left({\mu }^{\theta }+{\omega }^{\theta }\right),{m}_{8}={m}_{1}{m}_{3}{m}_{4}{m}_{6}{m}_{7}+{m}_{2}{m}_{4}{m}_{6}{m}_{7}{\varepsilon }^{\theta }{\Delta }^{\theta },\\ & {m}_{9}={m}_{3}{m}_{4}{m}_{6}{m}_{7}-{m}_{5}{\omega }^{\theta }{\gamma }^{\theta }-{m}_{6}{\sigma }^{\theta }{\omega }^{\theta }{\uppsi }^{\theta }, {m}_{10}={m}_{3}{m}_{4}{m}_{6}{m}_{7}{\mu }^{\theta }, {m}_{11}={m}_{4}{m}_{9}, \\ & {m}_{12}={m}_{4}{m}_{10}, {m}_{13}={m}_{3}{{m}_{4}}^{2}{{m}_{6}}^{2}{m}_{7}-{m}_{4}{m}_{5}{m}_{6}{\omega }^{\theta }{\gamma }^{\theta }-{m}_{4}{{m}_{6}}^{2}{\sigma }^{\theta }{\omega }^{\theta }{\uppsi }^{\theta }, {m}_{14}={m}_{3}{{m}_{4}}^{2}{{m}_{6}}^{2}{m}_{7}{\mu }^{\theta },\\ & {{m}_{15}=m}_{13}{\mu }^{\theta },\mathrm{ and }{m}_{16}={m}_{14}{\mu }^{\theta }.\end{aligned} $$

We substitute Eq. (16) with Eq. (17) into the expression ${\uplambda }_{n}^{*}\left(\mathrm{t}\right)=\frac{{\upbeta }^{\theta }}{{N}^{*}}\left({A}_{h}^{*}\left(\mathrm{t}\right)+{\varphi }^{\theta }{Q}_{h}^{*}\left(\mathrm{t}\right)\right)$ with

${N}^{*}={S}_{h}^{*}+{P}_{h}^{*}+{E}_{h}^{*}+{A}_{h}^{*}+{Q}_{h}^{*}+{R}_{h}^{*}$ and simplifying the computation we have carried out gives us

18 $${a}_{2}{{\uplambda }_{n}^{*}}^{2}+{ a}_{1}{\uplambda }_{n}^{*}+{ a}_{0}=0$$

where $${a}_{2}$$=$${\varepsilon }^{\theta }{\Delta }^{\theta }{m}_{4}{m}_{6}{m}_{7}{\mu }^{\theta }{m}_{9}{m}_{13}+{m}_{8}{m}_{6}{m}_{7}{m}_{3}{\mu }^{\theta }{m}_{13}+{m}_{5}{m}_{3}{m}_{8}{m}_{4}{m}_{6}{m}_{7}{\mu }^{\theta }{m}_{9}+{{m}_{3}m}_{5}{m}_{8}{\delta }^{\theta }{m}_{4}{m}_{6}{m}_{7}{m}_{9}$$+$${{{m}_{3}m}_{5}{\gamma }^{\theta }m}_{8}{\mu }^{\theta }{m}_{13}$$+$${{m}_{3}{{m}_{6}{\sigma }^{\theta }{\uppsi }^{\theta }m}_{8}{\mu }^{\theta }m}_{13}>0,$$

19 $$ \begin{aligned}{a}_{1}&={m}_{8}{{m}_{3}m}_{4}{m}_{6}{m}_{7}{\mu }^{\theta }{m}_{13}+{{\varepsilon }^{\theta }{\Delta }^{\theta }{m}_{4}{m}_{6}{m}_{7}{\mu }^{\theta }m}_{10}{m}_{13}+{\varepsilon }^{\theta }{\Delta }^{\theta }{m}_{4}{m}_{6}{m}_{7}{\mu }^{\theta }{m}_{9}{m}_{14}+{{{m}_{8}m}_{6}{m}_{7}{m}_{3}{\mu }^{\theta }m}_{14}\\ &\quad+{m}_{5}{m}_{3}{m}_{8}{m}_{4}{m}_{6}{m}_{7}{\mu }^{\theta }{m}_{10}+{{m}_{3}m}_{5}{m}_{8}{\delta }^{\theta }{m}_{4}{m}_{6}{m}_{7}{m}_{10}+{m}_{14}{{{m}_{3}m}_{5}{\gamma }^{\theta }m}_{8}{\mu }^{\theta }+{{m}_{3}{{m}_{6}{\sigma }^{\theta }{\uppsi }^{\theta }m}_{8}{\mu }^{\theta }m}_{14}\\ &\quad- {\beta }^{\theta }{{m}_{3}m}_{5}{m}_{8}{m}_{4}{m}_{6}{m}_{7}{\mu }^{\theta }{m}_{9}- {\beta }^{\theta }\varphi {m}_{3}{m}_{5}{m}_{8}\delta {m}_{4}{m}_{6}{m}_{7}{m}_{9}\end{aligned} $$

and

$${a}_{0}={\varepsilon }^{\theta }{\Delta }^{\theta }{m}_{4}{m}_{6}{m}_{7}{\mu }^{\theta }{m}_{10}{m}_{14}\left[1-{\mathcal{R}}_{eff}^{\theta }\right]>0\,\,\mathrm{ iff }\,\,{\mathcal{R}}_{eff}^{\theta }<1.$$

Since all the model parameters are non-negative one can be seen from Eqs. (17) and (19) that ${a}_{2}>0$. Moreover, ${a}_{0}>0$ whenever ${\mathcal{R}}_{eff}^{\theta }<1$. Thus, the number of possible positive real roots the degree two polynomial (Eq. 18) can have depends on the sign of ${a}_{1}$. We analyzed it using the Descartes' rule of signs on the quadratic polynomial $f(x) = { a}_{2}{x}^{2}+{ a}_{1}x+{ a}_{0}$ (with $x$ = ${\uplambda }_{n}^{*}$ ).

##### Theorem 5

*The higher institutions students anxiety towards mathematics fractional order model* (Eq. 12).

$(\mathrm{a}).$ has a unique endemic equilibrium if ${\mathcal{R}}_{eff}^{\theta }>1$ and either of the following holds,$\left(\mathrm{i}\right). { a}_{1}>0$,

$$(\mathrm{ii}).$$ $${a}_{1}<0$$,

$(\mathrm{b}).$ Could have two endemic equilibrium if ${\mathcal{R}}_{eff}^{\theta }<1$ whenever ${a}_{1}$ has negative sign .

Theorem 5 condition (b) suggests the possibility of existence of multiple endemic equilibriums whenever ${\mathcal{R}}_{eff}^{\theta }<1$ (which is typically shows the existence of the phenomenon of backward bifurcation see references^23,26–33^. The phenomenon of backward bifurcation is characterized by the co-existence of a stable anexiety-free equilibrium and a stable anxiety endemic equilibrium whenever the effective reproduction number of the model is less than unity.

### Local stability of the anxiety endemic equilibrium point of the model (Eq. 12)

#### Theorem 6

*Suppose* ${E}_{A}^{*}$ *be the anxiety endemic equilibrium point of the model* (*Eq*. 12) *given by* *Eq*. (16) *and stated in Theorem 5 then it is locally asymptotically stable*.

#### Proof

To prove the local asymptotic stability of the anxiety endemic equilibrium poin we can apply the fractional odrder Routh Hurwitz stability criteria stated in reference^34^ , it is enough to show that all eigenvalues of the following Jacobian matrix satisfy the Matignon condition^35^ stated in Theorem 3.

Let ${E}_{A}^{*}=({S}_{h}^{*},{P}_{h}^{*},{E}_{h}^{*},{A}_{h}^{*},{Q}_{h}^{*},{R}_{h}^{*})$ be the fractional order model (Eq. 12) anxiety endemic equilibrium point given in Eq. (16). Then the Jacobian matrix of the model (Eq. 12) is computed as

$$J\left({E}_{A}^{*}\right)=\left(\begin{array}{cccccc}-\left({\lambda }_{n}^{*}+{\mu }^{\theta }\right)& (1-{\upkappa }^{\theta }){\uprho }^{\theta }& 0& -{\upbeta }^{\theta }(\frac{{N}^{*}-{A}_{h}^{*}}{{{N}^{*}}^{2}}){S}_{h}^{*}& -{\upbeta }^{\theta }(\frac{{N}^{*}-{Q}_{h}^{*}}{{{N}^{*}}^{2}}){\mathrm{\varphi }}^{\theta }{S}_{h}^{*}& \omega \\ 0& -\left({\upmu }^{\theta }+(1-{\upkappa }^{\theta }){\rho }^{\theta }\right)& 0& 0& 0& 0\\ {\lambda }_{n}^{*}& 0& -\left({\mu }^{\theta }+{\uppsi }^{\theta }\right)& {\upbeta }^{\theta }(\frac{{N}^{*}-{A}_{h}^{*}}{{{N}^{*}}^{2}}){S}_{h}^{*}& {\upbeta }^{\theta }(\frac{{N}^{*}-{Q}_{h}^{*}}{{{N}^{*}}^{2}}){\mathrm{\varphi }}^{\theta }{S}_{h}^{*}& 0\\ 0& 0& \left(1-{\upsigma }^{\theta }\right){\uppsi }^{\theta }& -\left({\mu }^{\theta }+{\delta }^{\theta }+{\gamma }^{\theta }\right)& 0& 0\\ 0& 0& 0& {\delta }^{\theta }& -{\mu }^{\theta }& 0\\ 0& 0& {\upsigma }^{\theta }{\uppsi }^{\theta }& {\gamma }^{\theta }& 0& -({\mu }^{\theta }+{\omega }^{\theta })\end{array}\right)$$

Then the possible eigenvalues of $J\left({E}_{A}^{*}\right)$ are computed as${\lambda }_{1}=-\left({\upmu }^{\theta }+(1-{\upkappa }^{\theta }){\rho }^{\theta }\right)<0$ and,

20 $${\lambda }^{5}+{a}_{1}{\lambda }^{4}+{a}_{2}{\lambda }^{3}+{a}_{3}{\lambda }^{2}+{a}_{4}\lambda +{a}_{5}=0$$

where,

$${a}_{1}=\left({\lambda }_{n}^{*}+{\mu }^{\theta }\right)+\left({\mu }^{\theta }+{\omega }^{\theta }\right)+\left({\mu }^{\theta }+{\uppsi }^{\theta }\right)+\left({\mu }^{\theta }+{\delta }^{\theta }+{\gamma }^{\theta }\right){\mu }^{\theta }+\left({\mu }^{\theta }+{\delta }^{\theta }+{\gamma }^{\theta }\right)>0,$$

$$ \begin{aligned}{a}_{2}&=\left({\mu }^{\theta }+{\omega }^{\theta }\right)\left({\lambda }_{n}^{*}+{\mu }^{\theta }\right)+\left({\mu }^{\theta }+{\uppsi }^{\theta }\right)\left({\lambda }_{n}^{*}+{\mu }^{\theta }\right)+{\mu }^{\theta }\left({\lambda }_{n}^{*}+{\mu }^{\theta }\right)+\left({\mu }^{\theta }+{\delta }^{\theta }+{\gamma }^{\theta }\right)\left({\lambda }_{n}^{*} +{\mu }^{\theta }\right)\\ & \quad + \left({\mu }^{\theta }+{\uppsi }^{\theta }\right)\left({\mu }^{\theta }+{\omega }^{\theta }\right)+{\mu }^{\theta }\left({\mu }^{\theta }+{\omega }^{\theta }\right){\lambda }^{3}+\left({\mu }^{\theta }+{\delta }^{\theta }+{\gamma }^{\theta }\right)\left({\mu }^{\theta }+{\omega }^{\theta }\right)+\left({\mu }^{\theta }+{\uppsi }^{\theta }\right){\mu }^{\theta }\\ & \quad+\left({\mu }^{\theta }+{\uppsi }^{\theta }\right)\left({\mu }^{\theta }+{\delta }^{\theta }+{\gamma }^{\theta }\right)+{\upbeta }^{{\varvec{\theta}}}\left(1-{\upsigma }^{\theta }\right){\uppsi }^{\theta }\left(\frac{{N}^{*}-{A}_{h}^{*}}{{{N}^{*}}^{2}}\right){S}_{h}^{*}{\mu }^{\theta }{\lambda }^{2}\\ &\quad+{\upbeta }^{{\varvec{\theta}}}\left(1-{\upsigma }^{\theta }\right){\uppsi }^{\theta }\left(\frac{{N}^{*}-{A}_{h}^{*}}{{{N}^{*}}^{2}}\right){S}_{h}^{*}>0,\end{aligned} $$

$$ \begin{aligned}{a}_{3}&=\left({\mu }^{\theta }+{\uppsi }^{\theta }\right)\left({\mu }^{\theta }+{\omega }^{\theta }\right)\left({\lambda }_{n}^{*}+{\mu }^{\theta }\right)+\left({\mu }^{\theta }+{\omega }^{\theta }\right)\left({\lambda }_{n}^{*}+{\mu }^{\theta }\right){\mu }^{\theta }+\left({\mu }^{\theta }+{\delta }^{\theta }+{\gamma }^{\theta }\right)\left({\mu }^{\theta }+{\omega }^{\theta }\right)\left({\lambda }_{n}^{*}+{\mu }^{\theta }\right)\\ &\quad+\left({\mu }^{\theta }+{\uppsi }^{\theta }\right){\mu }^{\theta }\left({\lambda }_{n}^{*}+{\mu }^{\theta }\right)+\left({\mu }^{\theta }+{\uppsi }^{\theta }\right)\left({\mu }^{\theta }+{\delta }^{\theta }+{\gamma }^{\theta }\right)\left({\lambda }_{n}^{*}+{\mu }^{\theta }\right)+\left({\mu }^{\theta }+{\delta }^{\theta }+{\gamma }^{\theta }\right){\mu }^{\theta }\left({\lambda }_{n}^{*}+{\mu }^{\theta }\right)\\ &\quad+\left({\mu }^{\theta }+{\uppsi }^{\theta }\right){\mu }^{\theta }\left({\mu }^{\theta }+{\omega }^{\theta }\right)+\left({\mu }^{\theta }+{\uppsi }^{\theta }\right)\left({\mu }^{\theta }+{\delta }^{\theta }+{\gamma }^{\theta }\right)\left({\mu }^{\theta }+{\omega }^{\theta }\right)+\left({\mu }^{\theta }+{\delta }^{\theta }+{\gamma }^{\theta }\right){\mu }^{\theta }\\ &\quad\left({\mu }^{\theta }+{\omega }^{\theta }\right)+\left({\mu }^{\theta }+{\delta }^{\theta }+{\gamma }^{\theta }\right)\left({\mu }^{\theta }+{\uppsi }^{\theta }\right){\mu }^{\theta }+{\upbeta }^{\theta }\left(1-{\upsigma }^{\theta }\right){\uppsi }^{\theta }\left(\frac{{N}^{*}-{A}_{h}^{*}}{{{N}^{*}}^{2}}\right){S}_{h}^{*}\left({\lambda }_{n}^{*}+{\mu }^{\theta }\right)\\ &\quad+{\upbeta }^{\theta }\left(1-{\upsigma }^{\theta }\right){\uppsi }^{\theta }\left(\frac{{N}^{*}-{A}_{h}^{*}}{{{N}^{*}}^{2}}\right){S}_{h}^{*}\left({\mu }^{\theta }+{\omega }^{\theta }\right)+{\upbeta }^{\theta }\left(1-{\upsigma }^{\theta }\right){\uppsi }^{\theta }\left(\frac{{N}^{*}-{Q}_{h}^{*}}{{{N}^{*}}^{2}}\right){\delta }^{\theta }{\mathrm{\varphi }}^{\theta }{S}_{h}^{*}\\ &\quad+{\lambda }_{n}^{*}\left(1-{\upsigma }^{\theta }\right){\uppsi }^{\theta }{\upbeta }^{{\varvec{\theta}}}\left(\frac{{N}^{*}-{A}_{h}^{*}}{{{N}^{*}}^{2}}\right){S}_{h}^{*}+{\lambda }_{n}^{*}{\upsigma }^{\theta }{\uppsi }^{\theta }{\omega }^{\theta }>0,\end{aligned} $$

$$ \begin{aligned}{a}_{4} & =  \left({\mu }^{\theta }+{\uppsi }^{\theta }\right)\mu \left({\mu }^{\theta }+{\omega }^{\theta }\right)\left({\lambda }_{n}^{*}+{\mu }^{\theta }\right)+\left({\mu }^{\theta }+{\uppsi }^{\theta }\right)\left({\mu }^{\theta }+{\delta }^{\theta }+{\gamma }^{\theta }\right)\left({\mu }^{\theta }+{\omega }^{\theta }\right)\left({\lambda }_{n}^{*} +{\mu }^{\theta }\right)\\ & \quad +\left({\mu }^{\theta }+{\delta }^{\theta }+{\gamma }^{\theta }\right)\mu \left({\mu }^{\theta }+{\omega }^{\theta }\right)\left({\lambda }_{n}^{*}+{\mu }^{\theta }\right)+\left({\mu }^{\theta }+{\delta }^{\theta }+{\gamma }^{\theta }\right)\left({\mu }^{\theta }+{\uppsi }^{\theta }\right)\mu \left({\lambda }_{n}^{*}+{\mu }^{{\varvec{\theta}}}\right)\\ & \quad+\left({\mu }^{\theta }+{\delta }^{\theta }+{\gamma }^{\theta }\right)\left({\mu }^{\theta }+{\uppsi }^{\theta }\right)\mu \left({\mu }^{\theta }+{\omega }^{\theta }\right)+{\upbeta }^{{\varvec{\theta}}}\left(1-\upsigma \right)\uppsi \left({\mu }^{\theta }+{\omega }^{\theta }\right)\left({\lambda }_{n}^{*}+{\mu }^{\theta }\right)\left(\frac{{N}^{*}-{A}_{h}^{*}}{{{N}^{*}}^{2}}\right){S}_{h}^{*}\\ & \quad+{\upbeta }^{{\varvec{\theta}}}\left(1-\upsigma \right)\uppsi \left(\frac{{N}^{*}-{A}_{h}^{*}}{{{N}^{*}}^{2}}\right){S}_{h}^{*}\mu \left({\lambda }_{n}^{*}+{\mu }^{\theta }\right)+{\upbeta }^{{\varvec{\theta}}}\left(1-\upsigma \right)\uppsi \left(\frac{{N}^{*}-{Q}_{h}^{*}}{{{N}^{*}}^{2}}\right)\delta \mathrm{\varphi }{S}_{h}^{*}\left({\lambda }_{n}^{*}+\mu \right)\\ & \quad+{\upbeta }^{{\varvec{\theta}}}\left(1-\upsigma \right)\uppsi \left(\frac{{N}^{*}-{A}_{h}^{*}}{{{N}^{*}}^{2}}\right){S}_{h}^{*}\mu \left({\mu }^{\theta }+{\omega }^{\theta }\right)+{\upbeta }^{{\varvec{\theta}}}\left(1-\upsigma \right)\uppsi \left(\frac{{N}^{*}-{Q}_{h}^{*}}{{{N}^{*}}^{2}}\right)\delta \mathrm{\varphi }{S}_{h}^{*}\left(\mu +\omega \right)\\ & \quad +{\lambda }_{n}^{*}\left(1-\upsigma \right)\uppsi \omega \gamma \mu +{\lambda }_{n}^{*}\left(1-\upsigma \right)\uppsi \omega \gamma -{\lambda }_{n}^{*}\left(1-\upsigma \right)\uppsi {\upbeta }^{{\varvec{\theta}}}\left(\frac{{N}^{*}-{A}_{h}^{*}}{{{N}^{*}}^{2}}\right){S}_{h}^{*}\left({\mu }^{\theta }+{\omega }^{\theta }\right)\\ &\quad-{\upbeta }^{{\varvec{\theta}}}{\lambda }_{n}^{*}\left(1-\upsigma \right)\uppsi \left(\frac{{N}^{*}-{Q}_{h}^{*}}{{{N}^{*}}^{2}}\right)\delta \mathrm{\varphi }{S}_{h}^{*}-{\lambda }_{n}^{*}\mathrm{\sigma \psi }\omega \mu -{\lambda }_{n}^{*}\mathrm{\sigma \psi }\omega \left({\mu }^{\theta }+{\delta }^{\theta }+{\gamma }^{\theta }\right)>0\end{aligned} $$

$$ \begin{aligned}{a}_{5}&=\left({\mu }^{\theta }+{\delta }^{\theta }+{\gamma }^{\theta }\right)\left({\mu }^{\theta }+{\uppsi }^{\theta }\right)\mu \left({\mu }^{\theta }+{\omega }^{\theta }\right)\left({\lambda }_{n}^{*}+{\mu }^{\theta }\right)+{\upbeta }^{{\varvec{\theta}}}\left(1-\upsigma \right)\uppsi \left({\mu }^{\theta }+{\omega }^{\theta }\right)\left({\lambda }_{n}^{*}+{\mu }^{\theta }\right)\\ &\quad\left(\frac{{N}^{*}-{A}_{h}^{*}}{{{N}^{*}}^{2}}\right){S}_{h}^{*}\mu +{\upbeta }^{{\varvec{\theta}}}\left(1-\upsigma \right)\uppsi \left({\mu }^{\theta }+{\omega }^{\theta }\right)\left({\lambda }_{n}^{*}+{\mu }^{\theta }\right)\left(\frac{{N}^{*}-{Q}_{h}^{*}}{{{N}^{*}}^{2}}\right)\delta \mathrm{\varphi }{S}_{h}^{*}-{\lambda }_{n}^{*}\left(1-\upsigma \right)\uppsi \\ &\quad{\upbeta }^{{\varvec{\theta}}}\left(\frac{{N}^{*}-{A}_{h}^{*}}{{{N}^{*}}^{2}}\right){S}_{h}^{*}\left({\mu }^{\theta }+{\omega }^{\theta }\right)\mu -{\upbeta }^{{\varvec{\theta}}}{\lambda }_{n}^{*}\left(1-\upsigma \right)\uppsi \left(\frac{{N}^{*}-{Q}_{h}^{*}}{{{N}^{*}}^{2}}\right)\delta \mathrm{\varphi }{S}_{h}^{*}\left({\mu }^{\theta }+{\omega }^{\theta }\right)\\ &\quad-{\lambda }_{n}^{*}\mathrm{\sigma \psi }\omega \mu \left({\mu }^{\theta }+{\delta }^{\theta }+{\gamma }^{\theta }\right)>0.\end{aligned} $$

Here after a detailed comutations of the Routh-Hurwitz determinants of Eq. (19) gives us the following results

$${\Delta }_{1}={a}_{1}>0, {\Delta }_{2}=\left|\begin{array}{ll}{a}_{1}& 1\\ {a}_{3}& {a}_{2}\end{array}\right|={a}_{1}{a}_{2}-{a}_{3}>0, {\Delta }_{3}=\left|\begin{array}{ccc}{a}_{1}& 1& 0\\ {a}_{3}& {a}_{2}& {a}_{1}\\ {a}_{5}& {a}_{4}& {a}_{3}\end{array}\right|>0$$

$${\Delta }_{4}=\left|\begin{array}{llll}{a}_{1}& 1& 0& 0\\ {a}_{3}& {a}_{2}& {a}_{1}& 1\\ {a}_{5}& {a}_{4}& {a}_{3}& {a}_{2}\\ 0& 0& {a}_{5}& {a}_{4}\end{array}\right|>0,\mathrm{ and }{\Delta }_{4}=\left|\begin{array}{lllll}{a}_{1}& 1& 0& 0& 0\\ {a}_{3}& {a}_{2}& {a}_{1}& 1& 0\\ {a}_{5}& {a}_{4}& {a}_{3}& {a}_{2}& 0\\ 0& 0& {a}_{5}& {a}_{4}& 0\\ 0& 0& 0& 0& {a}_{5}\end{array}\right|>0$$

Since each ${a}_{i}{^{\prime}}s$ and ${\Delta }_{i}{^{\prime}}s$ for $i={1,2},{3,4},$ and $5$ are positive all the six eigenvalues of the Jacobian matrix $J\left({E}_{A}^{*}\right)$ are negative and which satisfies the Matignon condition^35^ stated in Theorem 3 ,i.e., $\left|{\lambda }_{i}\right|>\theta \frac{\pi }{2}$ for $0\le \theta <1$. Therefore, the anxiety endemic equilibrium point of the fractional order model (Eq. 12) is locally asymptotically stable.

#### Global asymptotic stability of the anxiety endemic equilibrium point

##### Theorem 7

*Let* $0<\theta <1$ *be the order of the fractional order model given in* (Eq. 12) *then the unique anxiety endemic equilibrium point* ${E}_{A}^{*}$ *whenever* ${\mathcal{R}}_{eff}^{\theta }>1$ *is globally asymptotically stable*.

##### Proof

Based on the Lyapnove function method stated in^23,36^, we can construct the following function $H\left(t\right)={H}_{1}\left({S}_{h}\right)+{H}_{2}\left({P}_{h}\right)+{H}_{3}\left({E}_{h}\right)+{H}_{4}\left({A}_{h}\right)+{H}_{5}\left({Q}_{h}\right)+{H}_{6}({R}_{h})$ where

$$ \begin{aligned} & {H}_{1}\left({S}_{h}(t)\right)=\frac{1}{2}{({S}_{h}-{S}_{h}^{*})}^{2}, {H}_{2}\left({P}_{h}(t)\right)=\frac{1}{2}{({P}_{h}-{P}_{h}^{*})}^{2}, {H}_{3}\left({E}_{h}(t)\right)=\frac{1}{2}{({E}_{h}-{E}_{h}^{*})}^{2},\\ & {H}_{4}\left({A}_{h}(t)\right)=\frac{1}{2}{({A}_{h}-{A}_{h}^{*})}^{2}, {H}_{5}\left({Q}_{h}(t)\right)=\frac{1}{2}{({Q}_{h}-{Q}_{h}^{*})}^{2},\mathrm{ and }{H}_{6}\left({R}_{h}(t)\right)=\frac{1}{2}{({R}_{h}-{R}_{h}^{*})}^{2}\end{aligned} $$

Since $H\left(t\right)$ is contionous the Caputo fractional order derivative of $H\left(t\right)$ is computed as.

$${^{\rm C}D}_{t}^{\theta }\mathrm{H}\left(\mathrm{t}\right)\le \left({S}_{h}-{S}_{h}^{*}\right){D}_{t}^{\theta }{S}_{h}+\left({P}_{h}-{P}_{h}^{*}\right){D}_{t}^{\theta }{P}_{h}+\left({E}_{h}-{E}_{h}^{*}\right){D}_{t}^{\theta }{E}_{h}+\left({A}_{h}-{A}_{h}^{*}\right){D}_{t}^{\theta }{A}_{h}+ \left({Q}_{h}-{Q}_{h}^{*}\right){D}_{t}^{\theta }{Q}_{h}+ \left({R}_{h}-{R}_{h}^{*}\right){D}_{t}^{\theta }{R}_{h}.$$

$$ \begin{aligned}B&=\left(\left(1-{\varepsilon }^{\theta }\right){\Delta }^{\theta }+{\omega }^{\theta }{R}_{h}+\left(1-{\upkappa }^{\theta }\right){\uprho }^{\theta }{P}_{h}-\left({\lambda }_{n}+{\mu }^{\theta }\right){S}_{h}\right){S}_{h}^{*}\\ &\quad+ ({\varepsilon }^{\theta }{\Delta }^{\theta }-\left({\mu }^{\theta }+\left(1-{\upkappa }^{\theta }\right){\rho }^{\theta }\right){P}_{h}{P}_{h}^{*}+( {\lambda }_{n}{\mathrm{S}}_{h}-\left({\mu }^{\theta }+{\psi }^{\theta }\right){E}_{h}){E}_{h}^{*}\\ &\quad+(\left(\left(1-{\sigma }^{\theta }\right)\psi {E}_{h}-\left({\mu }^{\theta }+{\delta }^{\theta }+{\gamma }^{\theta }\right){A}_{h}\right){A}_{h}^{*}+ ({\delta }^{\theta }{A}_{h}-{\mu }^{\theta }{Q}_{h}){Q}_{h}^{*}\\ &\quad+\left({A}_{h}+{\psi }^{\theta }{\sigma }^{\theta }{E}_{h}-\left({\mu }^{\theta }+{\omega }^{\theta }\right){R}_{h}\right){R}_{h}^{*}>0.\end{aligned} $$

Then after solving this expression we have determined the following result

$$\Rightarrow $$ ^C^$${D}_{t}^{\theta }\mathrm{H}\left(\mathrm{t}\right)\le A-B$$ where

$$A=\left(\left(1-{\varepsilon }^{\theta }\right){\Delta }^{\theta }+{\omega }^{\theta }{R}_{h}+\left(1-{\upkappa }^{\theta }\right){\uprho }^{\theta }{P}_{h}-\left({\lambda }_{n}+{\mu }^{\theta }\right){{S}_{h}}^{2}\right)+ ({\varepsilon }^{\theta }{\Delta }^{\theta }-\left({\mu }^{\theta }+\left(1-{\upkappa }^{\theta }\right){\rho }^{\theta }\right){{P}_{h}}^{2}+ \left( {\lambda }_{n}{\mathrm{S}}_{h}-\left({\mu }^{\theta }+{\psi }^{\theta }\right){E}_{h}\right){E}_{h}+ \left(\left(1-{\sigma }^{\theta }\right){\psi }^{\theta }{E}_{h}-\left({\mu }^{\theta }+{\delta }^{\theta }+{\gamma }^{\theta }\right){A}_{h}\right){A}_{h}+ \left({\delta }^{\theta }{A}_{h}-{\mu }^{\theta }{Q}_{h}\right){Q}_{h}+\left({A}_{h}+{\psi }^{\theta }{\sigma }^{\theta }{E}_{h}-\left({\mu }^{\theta }+{\omega }^{\theta }\right){R}_{h}\right){R}_{h}>0$$

and

$$B=\left(\left(1-{\varepsilon }^{\theta }\right){\Delta }^{\theta }+{\omega }^{\theta }{R}_{h}+\left(1-{\upkappa }^{\theta }\right){\uprho }^{\theta }{P}_{h}-\left({\lambda }_{n}+{\mu }^{\theta }\right){S}_{h}\right){S}_{h}^{*}+ ({\varepsilon }^{\theta }{\Delta }^{\theta }-\left({\mu }^{\theta }+\left(1-{\upkappa }^{\theta }\right){\rho }^{\theta }\right){P}_{h}{P}_{h}^{*}+( {\lambda }_{n}{\mathrm{S}}_{h}-\left({\mu }^{\theta }+{\psi }^{\theta }\right){E}_{h}){E}_{h}^{*}+(\left(\left(1-{\sigma }^{\theta }\right)\psi {E}_{h}-\left({\mu }^{\theta }+{\delta }^{\theta }+{\gamma }^{\theta }\right){A}_{h}\right){A}_{h}^{*}+ ({\delta }^{\theta }{A}_{h}-{\mu }^{\theta }{Q}_{h}){Q}_{h}^{*}+\left({A}_{h}+{\psi }^{\theta }{\sigma }^{\theta }{E}_{h}-\left({\mu }^{\theta }+{\omega }^{\theta }\right){R}_{h}\right){R}_{h}^{*}>0.$$

Thus, ${D}_{t}^{\theta }\mathrm{H}\left(\mathrm{t}\right)\le 0$ if and only if the condition $A<B$ holds, and ${D}_{t}^{\theta }\mathrm{H}\left(\mathrm{t}\right)=0$ whenever $A=B$ or ${S}_{h}={S}_{h}^{*}$,${P}_{h}={P}_{h}^{*}$,${E}_{h}={E}_{h}^{*}$,${A}_{h}={A}_{h}^{*}$,${Q}_{h}={Q}_{h}^{*}$ and ${R}_{h}={R}_{h}^{*}$. Hence, by^22,23^ the function $\mathrm{H}$ is a Lyapnov function on the feasible domain and the largest set in this feasible domain satisfies the condition $\{({S}_{h},{P}_{h},{E}_{h},{A}_{h},{Q}_{h},{R}_{h})\in {\mathbb{R}}_{+}^{6},\mathrm{C}{D}_{t}^{\theta }\mathrm{H}=0\}$ is only the singleton set $\{\left({S}_{h}^{*},{P}_{h}^{*},{E}_{h}^{*},{A}_{h}^{*},{Q}_{h}^{*},{R}_{h}^{*}\right)\}$. Therefore, the anxiety endemic equilibrium point is globally asymptotically stable if all these conditions and ${\mathcal{R}}_{eff}^{\theta }>1$ holds.

## Optimal control problem

In this section, we extend the fractional order model (Eq. 12) by introducing two time-dependent controlling strategies, where ${c}_{1}\left(t\right)$ represents efforts to prevent students from anxiety infection and help reduce anxiety contact rates and ${c}_{2}\left(t\right)$ represents the intensity of educational treatment of mathematics anxious students to increase recovery from anxiety where 0 $\le {c}_{1}\left(t\right),{c}_{2}\left(t\right)\le 1$ . It is assumed that the exposed population in susceptible students is reduced by the factor $(1-$ ${c}_{1}\left(t\right)$) due to the protection measures taken. Similarly, the anxiety infected students is reduced by the factor $(1-$ ${c}_{2}\left(t\right)$) due to the educational treatment by experts. Hence, the control theory dynamical system of the Eq. (12) becomes:

$$^{\rm C}{D}_{t}^{\theta }{S}_{h}= (1-{\varepsilon }^{\theta }){\Delta }^{\theta }+{\omega }^{\theta }{R}_{h}+(1-{\upkappa }^{\theta }){\uprho }^{\theta }{P}_{h}-\left((1- {c}_{1}\left(t\right)) {\lambda }_{n}+{\mu }^{\theta }\right){S}_{h}$$

$$^{\rm C}{D}_{t}^{\theta }{P}_{h}={\varepsilon }^{\theta }{\Delta }^{\theta }-\left({\mu }^{\theta }+(1-{\upkappa }^{\theta }){\rho }^{\theta }\right){P}_{h}$$

21 $$^{\rm C}{D}_{t}^{\theta }{E}_{h}=(1-{c}_{1}\left(t\right)){\lambda }_{n}{\mathrm{S}}_{h}-\left({\mu }^{\theta }+{\psi }^{\theta }\right){E}_{h}$$

$$^{\rm C}{D}_{t}^{\theta }{A}_{h}= (1-{\sigma }^{\theta }){\psi }^{\theta }{E}_{h}-\left({\mu }^{\theta }+{\delta }^{\theta }+{c}_{2}\left(t\right){\gamma }^{\theta }\right){A}_{h}$$

$$^{\rm C}{D}_{t}^{\theta }{Q}_{h}={\delta }^{\theta }{A}_{h}-{\mu }^{\theta }{Q}_{h}$$

$$^{\rm C}{D}_{t}^{\theta }{R}_{h}={c}_{2}\left(t\right)\gamma {A}_{h}+{\psi }^{\theta }{\sigma }^{\theta }{E}_{h}-\left({\mu }^{\theta }+{\omega }^{\theta }\right){R}_{h}$$

with initial conditions ${S}_{h}\left(0\right)>0$,${P}_{h}\left(0\right)\ge 0, {E}_{h}\left(0\right)\ge 0, {A}_{h}\left(0\right)\ge 0$,${Q}_{h}\left(0\right)\ge 0$, and ${R}_{h}\left(0\right)\ge 0,$ and thecontrol set is ${\Omega }_{C}=\left\{{c}_{1}\left(t\right), {c}_{2}\left(t\right):0\le {c}_{1}\left(t\right), {c}_{2}\left(t\right)\le 1 , t\in \left[0,T\right]\right\}$, where $T$ is the final time of implementing controls.

To minimize the number of mathematics anxious students in the community we construct the objective function given by

22 $$J({c}_{1}, {c}_{2}) ={\int }_{0}^{T}\left({\mho }_{1}{E}_{h}+{\mho }_{2} {\mathrm{A}}_{h}+\frac{{\mathfrak{B}}_{1}}{2}{c}_{1}^{2}+\frac{{\mathfrak{B}}_{2}}{2}{c}_{2}^{2}\right)dt$$

The control problem involves a situation in which the number of mathematics anxiety infected students and the cost of applying preventions and treatments controls ${u}_{1}\left(t\right)$ and $u\left(t\right)$ are minimized subject to the system (29). Where $T$ is the final time, the coefficients ${\mho }_{1}$ and ${\mho }_{2}$ are positive weight constants and $\frac{{\mathfrak{B}}_{1}}{2}$ and $\frac{{\mathfrak{B}}_{2}}{2}$ are the measure of relative costs of interventions associated with the controls ${c}_{1}$ and ${c}_{2}$, respectively, and also balances the units of integrand. The objective is to find the optimal values ${C}^{*}=\left({c}_{1}^{*}, {c}_{2}^{*}\right)$ of the controls $C=\left({c}_{1}, {c}_{2}\right)$ such that the associated state trajectories ${S}_{h}^{*},{P}_{h}^{*}, {E}_{h}^{*}, {A}_{h}^{*}, {Q}_{h}^{*}, {R}_{h}^{*}$ are solution of the system (Eq. 22) in the intervention time interval $\left[0, T\right]$ with initial the given conditions and minimize the objective functional. In the cost functional, the term ${\mho }_{1}{E}_{h}$ refer to the cost related to anxiety exposed students and the term ${\mho }_{2} {\mathrm{A}}_{h}$ refer to the cost related to anxiety infected class.

### Theorem 8 (Existence of optimal solution)

*There exists an optimal control* ${C}^{*}=\left({c}_{1}^{*}, {c}_{2}^{*}\right)$ *in* ${\Omega }_{C}$ *and a corresponding solution vector* $\left({S}_{h}^{*},{P}_{h}^{*}, {E}_{h}^{*}, {A}_{h}^{*}, {Q}_{h}^{*}, {R}_{h}^{*}\right)$ *to the dynamical system* (Eq. 22) *with the given initial conditions such that* $J\left({c}_{1}^{*}, {c}_{2}^{*}\right)=\underset{{\Omega }_{\mathfrak{M}}}{\mathrm{min}}J\left({c}_{1}, {c}_{2}\right)$.

Note: We utilize Pontryagin's maximal principle stated in^3,37^ to determine the prerequisites for the optimal control model (Eq. 22). For the optimal control problem (Eq. 22) we define the Hamiltonian (H) function by

23 $$ \begin{aligned}\mathcal{H} & =  {\mho }_{1}{E}_{h}+{\mho }_{2} {\mathrm{A}}_{h}+\frac{{\mathfrak{B}}_{1}}{2}{c}_{1}^{2}+\frac{{\mathfrak{B}}_{2}}{2}{c}_{2}^{2}+{\lambda }_{1}\left((1-{\varepsilon }^{\theta }){\Delta }^{\theta }+{\omega }^{\theta }{R}_{h}+{\uprho }^{\theta }(1-{\upkappa }^{\theta }){P}_{h}\right. \\&\quad \left.-\left(\left(1-{c}_{1}\right){\lambda }_{n}+{\mu }^{\theta }\right){S}_{h}\right)+ {\lambda }_{2}\left({\varepsilon }^{\theta }{\Delta }^{\theta }-\left({\mu }^{\theta }+(1-{\kappa }^{\theta }\right){\rho }^{\theta }){P}_{h}\right)+ {\lambda }_{3}\left(\left(1-{c}_{1}\right){\lambda }_{n}{\mathrm{S}}_{h}\right. \\&\quad \left.-\left({\mu }^{\theta }+{c}_{2}{\upsigma }^{\theta }{\psi }^{\theta }+\left(1-{\upsigma }^{\theta }\right){\uppsi }^{\theta }\right){E}_{h}\right)+ {\lambda }_{4}\left(\left(1-{\upsigma }^{\theta }\right){\uppsi }^{\theta }{\mathrm{E}}_{h}-\left({\upmu }^{\theta }+{\updelta }^{\theta }+{c}_{2}{\upgamma }^{\theta }\right){A}_{h}\right)\\ &\quad+ {\lambda }_{5}\left({\delta }^{\theta }{A}_{h}-{\mu }^{\theta }{Q}_{h}\right)+ {\lambda }_{6}\left({c}_{2}{\gamma }^{\theta }{A}_{h}+{c}_{2}{\sigma }^{\theta }{\psi }^{\theta }{E}_{h}-({\mu }^{\theta }+{\omega }^{\theta }){R}_{h}\right)\end{aligned} $$

where ${\lambda }_{1}\left(t\right), {\lambda }_{2}\left(t\right), {\lambda }_{3}\left(t\right), {\lambda }_{4}\left(t\right), {\lambda }_{5}\left(t\right)$ and ${\lambda }_{6}\left(t\right)$ are the co-state variables or adjoint variables. Using the same method stated in^37^ for fractional order model approach we have determined the following:

$$-\frac{d{\lambda }_{1}}{dt}=\frac{\partial \mathcal{H}}{\partial {S}_{h}}=-{\lambda }_{1}\left(\left(1- {c}_{1}\left(t\right)\right){\lambda }_{n}+{\mu }^{\theta }\right),$$

24 $$ \begin{aligned}-\frac{d{\lambda }_{2}}{dt}&=\frac{\partial \mathcal{H}}{\partial {P}_{h}}=-{\lambda }_{1}{\uprho }^{\theta }\left(1-{\upkappa }^{\theta }\right)-{\lambda }_{2}\left({\mu }^{\theta }+\left(1-{\upkappa }^{\theta }\right){\rho }^{\theta }\right)-\frac{d{\lambda }_{3}}{dt}=\frac{\partial \mathcal{H}}{\partial {E}_{h}}\\ &=-{\mathrm{\mho }}_{1}-{\lambda }_{3}\left({\mu }^{\theta }+{c}_{2}{\upsigma }^{\theta }{\psi }^{\theta }+\left(1-{\upsigma }^{\theta }\right){\uppsi }^{\theta }\right)-{\lambda }_{4}\left(1-{\upsigma }^{\theta }\right){\uppsi }^{\theta }-{\lambda }_{6}{c}_{2}{\sigma }^{\theta }{\psi }^{\theta }-\frac{d{\lambda }_{4}}{dt}\\ &=\frac{\partial \mathcal{H}}{\partial {A}_{h}}=-{\mathrm{\mho }}_{2}-{\lambda }_{1}\left(1-{c}_{1}\right)\frac{{\upbeta }^{\theta }\left(N-{A}_{h}\right){S}_{h}}{{N}^{2}}-{\lambda }_{3}\left(1-{c}_{1}\right)\frac{{\upbeta }^{\theta }\left(N-{A}_{h}\right){S}_{h}}{{N}^{2}}\\ &\quad-{\lambda }_{4}\left({\upmu }^{\theta }+{\updelta }^{\theta }+{c}_{2}{\upgamma }^{\theta }\right)-{\lambda }_{5}{\updelta }^{\theta }-{\lambda }_{6}{c}_{2}{\gamma }^{\theta },-\frac{d{\lambda }_{5}}{dt}=\frac{\partial \mathcal{H}}{\partial {Q}_{h}}=-{\lambda }_{5}{\mu }^{\theta },-\frac{d{\lambda }_{6}}{dt}\\ &=\frac{\partial \mathcal{H}}{\partial {R}_{h}}=-{\lambda }_{1}{\omega }^{\theta }-{\lambda }_{6}\left({\mu }^{\theta }+{\omega }^{\theta }\right).\end{aligned} $$

The transversality conditions are

$${\lambda }_{i}^{*}\left(T\right)=0, i=1, 2, \dots ,6.$$

On the interior of the control set, where $0<{c}_{i}<1$ for $i={1,2}$ we have the following equations

$$\frac{\partial \mathcal{H}}{\partial {c}_{1}}=2{\mathfrak{B}}_{1}{c}_{1}+{\lambda }_{1}{\lambda }_{n}{\mathrm{S}}_{h}-{\lambda }_{3}{\lambda }_{n}{\mathrm{S}}_{h}{\mathrm{E}}_{h}=0,$$

$\frac{\partial \mathcal{H}}{\partial {c}_{2}}=2{\mathfrak{B}}_{2}{c}_{2}+\left({\lambda }_{6}-{\lambda }_{3}\right){\upsigma }^{\theta }{\psi }^{\theta }{\mathrm{E}}_{h}+\left({\lambda }_{6}-{\lambda }_{3}\right){\upgamma }^{\theta }{\mathrm{A}}_{h}=0.$ Then solving for for ${c}_{1}$ and ${c}_{2}$ gives us

$${c}_{1}=\frac{({\lambda }_{3}{\mathrm{E}}_{h}-{\lambda }_{1}){\lambda }_{n}{\mathrm{S}}_{h}}{2{\mathfrak{B}}_{1}},$$

$${c}_{2}=\frac{\left({\lambda }_{3}-{\lambda }_{6}\right){[\upsigma }^{\theta }{\psi }^{\theta }{\mathrm{E}}_{h}+{\upgamma }^{\theta }{\mathrm{A}}_{h}]}{2{\mathfrak{B}}_{2}}.$$

### Theorem 9

*The control parameters* (${c}_{1}^{*},{c}_{2}^{*}$) *that minimizes* $J({c}_{1}, {c}_{2})$ *are given by*:

$${c}_{1}^{*}\left(t\right)=\mathrm{max}\left\{0,\mathrm{ min}[1,\frac{\left({\lambda }_{3}{\mathrm{E}}_{h}-{\lambda }_{1}\right){\lambda }_{n}{\mathrm{S}}_{h}}{2{\mathfrak{B}}_{1}}]\right\},$$

$${c}_{2}^{*}\left(t\right)=\mathrm{max}\left\{0,\mathrm{min}[1, \frac{\left({\lambda }_{3}-{\lambda }_{6}\right){[\upsigma }^{\theta }{\psi }^{\theta }{\mathrm{E}}_{h}+{\upgamma }^{\theta }{\mathrm{A}}_{h}]}{2{\mathfrak{B}}_{2}}\right\},$$

*where* ${\lambda }_{1}\left(t\right), {\lambda }_{2}\left(t\right), {\lambda }_{3}\left(t\right), {\lambda }_{4}\left(t\right), {\lambda }_{5}\left(t\right)$ *and* ${\lambda }_{6}\left(t\right)$ *are the co-state variables or adjoint variables satisfying* (Eq. 24) *and the transversality conditions stated above*.

Then we have the following conditions$${c}_{1}^{*}=\left\{\begin{array}{l}0, if {c}_{1}\le 0\\ {c}_{1}, if 0<{c}_{1}<1\\ 1, if {c}_{1}>1\end{array}\right.$$, and

$${c}_{1}^{*}=\left\{\begin{array}{l}0, if {c}_{1}\le 0\\ {c}_{1}, if 0<{c}_{1}<1\\ 1, if {c}_{1}>1.\end{array}\right.$$

### Proof

Applying Pontryagin's maximal principle stated in^37^ we obtained the following results

$${D}_{t}^{\theta }{\lambda }_{1}=\frac{\partial \mathcal{H}}{\partial {S}_{h}}, {D}_{t}^{\theta }{\lambda }_{2}=\frac{\partial \mathcal{H}}{\partial {P}_{h}}, {D}_{t}^{\theta }{\lambda }_{3}=\frac{\partial \mathcal{H}}{\partial {E}_{h}},$$

$${D}_{t}^{\theta }{\lambda }_{4}=\frac{\partial \mathcal{H}}{\partial {A}_{h}}, {D}_{t}^{\theta }{\lambda }_{5}=\frac{\partial \mathcal{H}}{\partial {Q}_{h}}, {D}_{t}^{\theta }{\lambda }_{6}=\frac{\partial \mathcal{H}}{\partial {R}_{h}},$$

with transversality conditions ${\lambda }_{i}^{*}\left(T\right)=0$, $i=1, 2, \dots ,6$.

The optimality conditions is obtained by differentiating Hamiltonian $\mathcal{H}$ with respect to the control variables ${c}_{1}$ and ${c}_{2}$: $\frac{\partial \mathcal{H}}{\partial {c}_{1}}=0$ =,$\frac{\partial \mathcal{H}}{\partial {c}_{2}}=0.$

## Numerical simulations for the deterministic model (Eq. 9)

To illustrate the numerical results of the integer order model (Eq. 9), we consider the fixed parameters values some of them are stated in reference^3^ and some of them are assumed and given by $\Delta$ = 100, $\mu$= 0.5, $\psi$= 0.04,$\varepsilon$ = 0.4, $\kappa =0.8,$ $\gamma$= 0.01, $\upomega$ = 0.03, $\rho$ = 0.2,$\delta =0.3,$ $\sigma =0.04 ,$ $\varphi =1.3$, and $\beta$ = 1.4 where some of these values are taken from the animosity study in reference^3^. In this section we execute a numerical simulation of the higher institution students anxiety towards mathematics model (Eq. 3) to justify the analytical results we performed in “Model derivation in Caputo fractional order derivative approach” using Matlab standard ordinary diferential equations (ODEs) solver function ode^45^.

### Behaviour of solutions of the model (Eq. 3)

**Figure 2:**
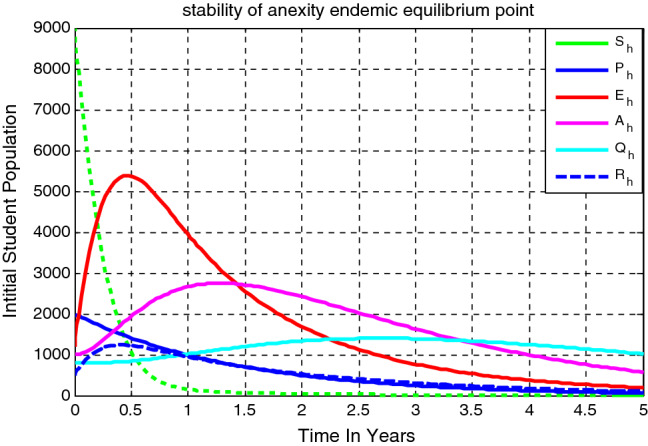
Model solutions trajectory over time (${\mathcal{R}}_{eff}>1).$

Figure 2 gives the higher institution students anxiety towards mathematics model (Eq. 13) solutions trajectory simulation where $\Delta = 100, \mu = 0.5, \psi = 0.04, \varepsilon = 0.4, \kappa =0.8, \gamma = 0.01,\upomega = 0.03, \rho = 0.2, \delta =0.3, \sigma =0.04 , \varphi =1.3,\mathrm{ and }\beta = 1.4,$ the model effective reproduction number is ${\mathcal{R}}_{eff}=5.41$. We can see that the higher institution students anxiety towards mathematics persists in the community and stabilizes in time. This means that the solution trajectories converging to the anxiety infection endemic equilibrium point.

### Effect of $\beta$ on ${\mathcal{R}}_{eff}$

**Figure 3:**
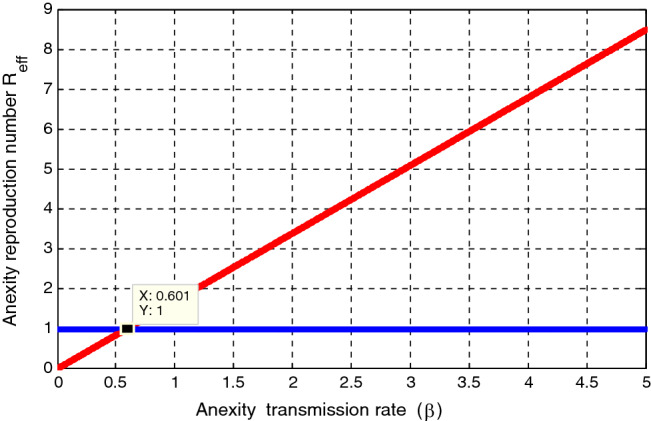
Effect of transmission rate $\beta$ on ${\mathcal{R}}_{eff}.$

Using parameter values $\Delta = 100, \mu = 0.5, \psi = 0.04, \varepsilon = 0.4, \kappa =0.8, \gamma = 0.01,\upomega = 0.03, \rho = 0.2, \delta =0.3, \sigma =0.04 , \varphi =1.3,\mathrm{ and }\beta = 1.4$, simulation in Fig. 3 investigated the effect of anxiety transmission rate $\beta$ on the anxiety effective reproduction number ${\mathcal{R}}_{eff}$. The figure expresses that when the value of the anxiety transmission rate $\beta$ increases, the anxiety effective reproduction number increases, and whenever the value of $\beta <0.601$ implies that ${\mathcal{R}}_{eff}<1.$ Therefore, the responsible body shall concentrate on minimizing the value of the anxiety transmission rate $\beta$ to prevent and control anxiety transmission in the higher institution community.

### Effect of ${\varvec{\gamma}}$ on ${\mathcal{R}}_{{\varvec{e}}{\varvec{f}}{\varvec{f}}}$

**Figure 4:**
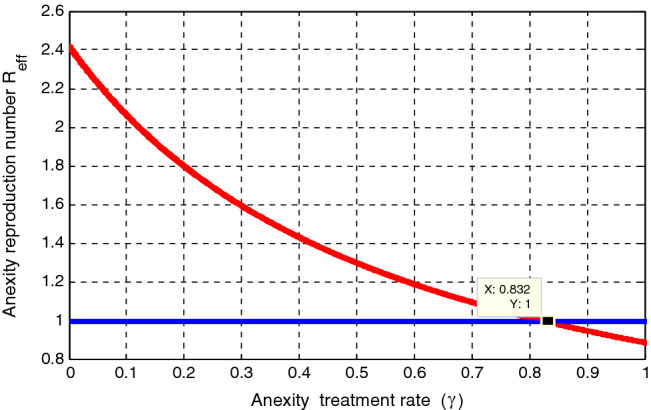
Effect of treatment rate $\gamma$ on ${\mathcal{R}}_{eff}$.

Using $\Delta = 100, \mu = 0.5, \psi = 0.04, \varepsilon = 0.4, \kappa =0.8, \gamma = 0.01,\upomega = 0.03, \rho = 0.2, \delta =0.3, \sigma =0.04 , \varphi =1.3,\mathrm{ and }\beta = 1.4$, simulation gieven by Fig. 4 illustrated that the effect of anxiety towards mathematics treatment rate $\gamma$ on the anxiety effective reproduction number ${\mathcal{R}}_{eff}$. The figure shows that when the value of the anxiety treatment rate $\gamma$ increases, the anxiety effective reproduction number deacreases, and whenever the value of $\gamma >0.832$ implies that ${\mathcal{R}}_{eff}<1.$ Therefore, the concerned body shall concentrate on maximizing the values of anxiety treatment rate $\gamma$ to minimize anxiety spreading throughout the student community.

### Effect of $\upvarepsilon$ on ${\mathcal{R}}_{eff}$

**Figure 5:**
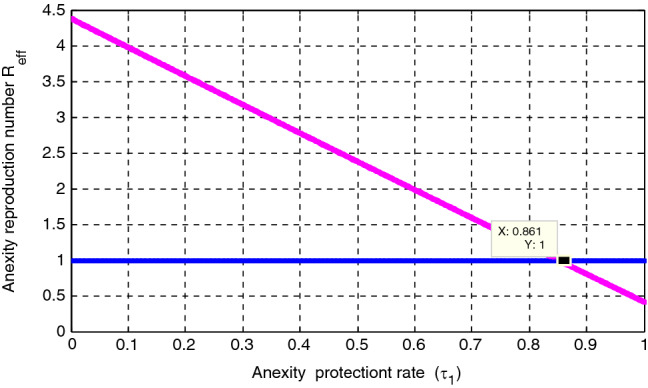
Effect of protection rate $\varepsilon$ on ${\mathcal{R}}_{eff}$.

In this subsection, using $\Delta = 100, \mu = 0.5, \psi = 0.04, \varepsilon = 0.4, \kappa =0.8, \gamma = 0.01,\upomega = 0.03, \rho = 0.2, \delta =0.3, \sigma =0.04 , \varphi =1.3,\mathrm{ and }\beta = 1.4 ,$ simulation gieven by Fig. 5 illustrated that the effect of anxiety towards mathematics protection rate $\varepsilon$ on the anxiety effective reproduction number ${\mathcal{R}}_{eff}$. The figure shows that when the value of the anxiety protectiont rate $\varepsilon$ increases, the anxiety effective reproduction number deacreases, and whenever the value of $\varepsilon >0.861$ implies that ${\mathcal{R}}_{eff}<1.$ Therefore, the concerned body shall concentrate on maximizing the values of anxiety protection rate $\gamma$ to minimize anxiety spreading throughout the student community.

### Effect of treatment rate ${\varvec{\gamma}}$ on the anxiety infectious students ${{\varvec{A}}}_{{\varvec{h}}}$

**Figure 6:**
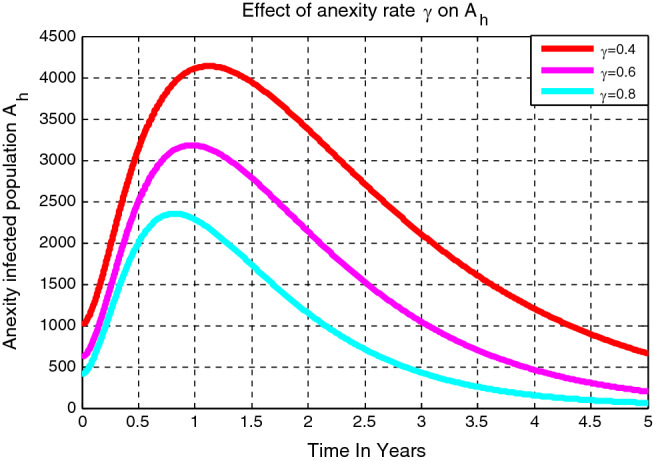
Effect of anxiety treatment rate $\gamma$ on ${A}_{h}.$

In this subsection, using $\Delta = 100, \mu = 0.5, \psi = 0.04, \varepsilon = 0.4, \kappa =0.8, \gamma = 0.01,\upomega = 0.03, \rho = 0.2, \delta =0.3, \sigma =0.04 , \varphi =1.3,\mathrm{ and }\beta = 1.4 ,$ simulation gieven by Fig. 6 illustrated that the effect of anxiety towards mathematics treatment rate $\gamma$ on the anxiety infectious student population ${A}_{h}$. The figure shows that when the value of the anxiety treatment rate $\gamma$ increases, the anxiety infectious student population ${A}_{h}$ deacreases.Therefore, the concerned body shall concentrate on maximizing the values of anxiety protection rate $\gamma$ to minimize anxiety infectious student population.

## Numerical simulations of the fractional order model (Eq. 12)

In this section the numerical simulation of the Caputo fractional order model (Eq. 12) is simulated by using fractional Euler’s forward method. To illustrate the numerical results of the fractional order model (Eq. 12), we considerd two possibilities for the order of the derivative as $\theta =0.5$ and $\theta =1$ and we have taken the fixed parameters used in the simulation of the integer order model (Eq. 9) with values $\Delta$ = 100, $\mu$= 0.5, $\psi$= 0.04,$\varepsilon$ = 0.4, $\kappa =0.8,$ $\gamma$= 0.01, $\upomega$ = 0.03, $\rho$ = 0.2,$\delta =0.3,$ $\sigma =0.04 ,$ $\varphi =1.3$, and $\beta$ = 1.4 where some of these values are related to animosity study in^3^. In this section we execute a numerical simulation of the higher institution students anxiety towards mathematics model (Eq. 12) to justify the analytical results we performed in “Model derivation in Caputo fractional order derivative approach” using Euler forward method and writing a Matlab code for the fractional order diferential equations (FODEs) given in (Eq. 12). In order to observe the effects that the parameter $\theta$ has on the dynamics of the fractional-order model (Eq. 12), we include several numerical simulations varying the value of this parameter.

### Effect of memory on higher institutions students anxiety infection

**Figure 7:**
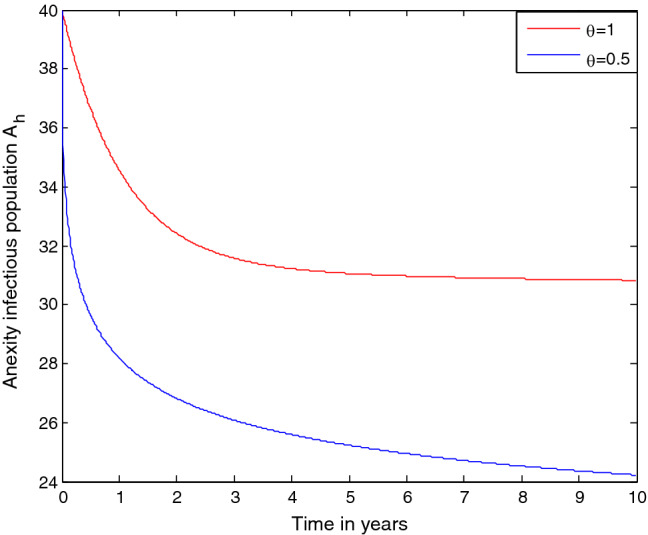
Order of derivative( memory) effects on anxiety infectious population ${A}_{h}$.

**Figure 8:**
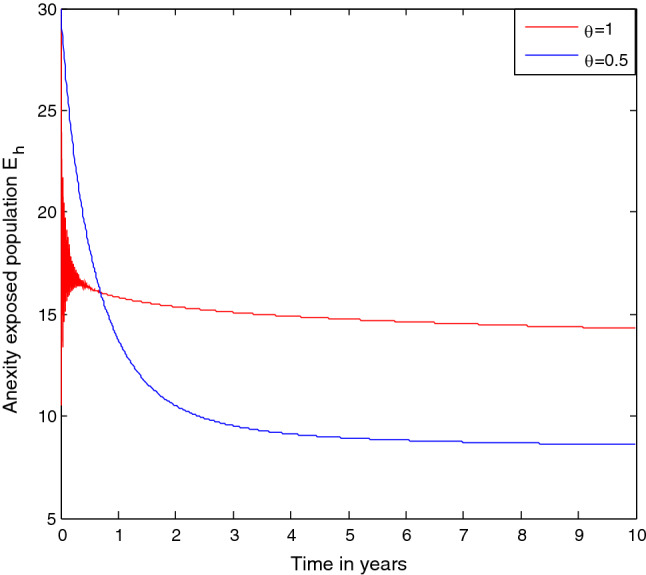
Order of derivative( memory) effects on anxiety exposed population ${E}_{h}$.

**Figure 9:**
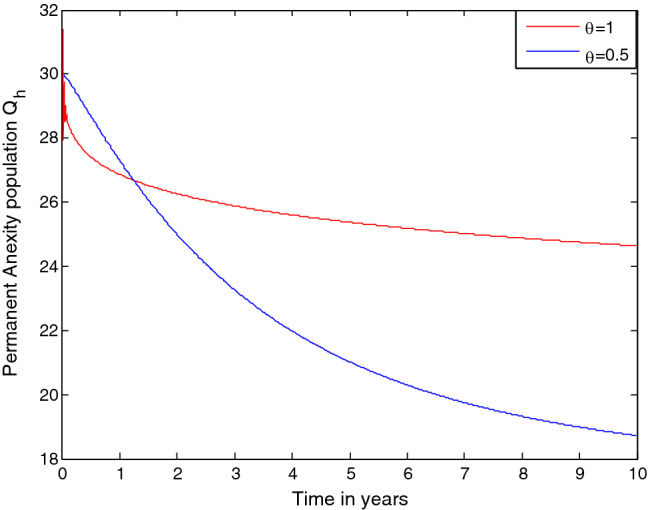
Order of derivative( memory) effects on permanent anxiety infectious population ${Q}_{h}$.

**Figure 10:**
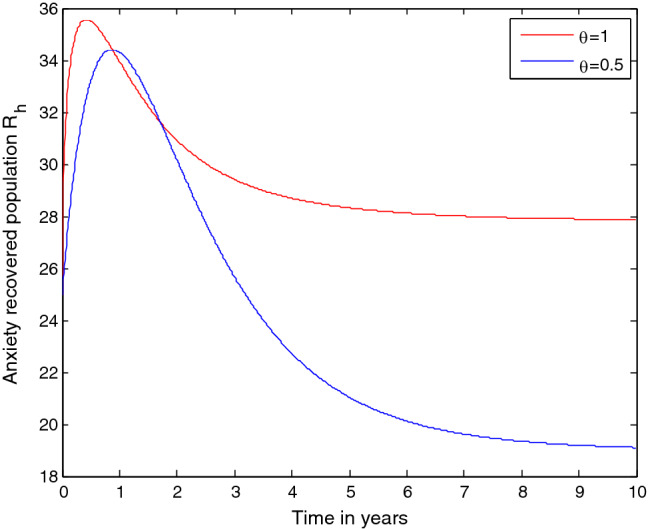
Order of derivative( memory) effects on anxiety recovered population ${R}_{h}$.

Here, we simulate the order of derivative (memory) effects ($\theta$) on the number of anxiety exposed, infected and recovered higher institutions students. In the simulations given in Figs. 7, 8, 9 and 10, we compare the number of anxiety exposed, infected and recovered higher institutions students for memory-less(integer order) model that is when ($\theta =1$) and model with memory that is when ($\theta =0.5$). We illustrated the effects of memory(order of derivatives)($\theta$) on the number of anxiety exposed, infected and recovered higher institutions students from Figs. 7, 8, 9 and 10. As we observe in Fig. 7, the number of anxiety infectious students ${(A}_{h})$ is larger in the system with out memory ($\theta =1$) as compared with system with memory ($\theta =0.5$). Similarly from Figs. 8, 9 and 10 we observed that the number of anxiety exposed ${(E}_{h}) ,$ permanently anxitious ${(Q}_{h})$ and anexiety recovered ${(R}_{h})$ are larger in system with out memory ($\theta =1$) compared with systems with memory ($\theta =0.5$). In this section computation of the model (Eq. 12) effective reproduction number is determined as ${\mathcal{R}}_{eff}^{\theta }=2.83$ which shows the persistence of mathematics anxiety in the student community.

### Effect of optimal control strategies on higher institutions students mathematics anxiety

In this sub-section we have carried out the numerical simulation of the state variables in the optimal control problem given in Eq. (21) to investigate the effect of controlling strategies using Euler forward method where the order of the derivative is assumed to be $\theta =0.5$ and some of the fixed parameters values given in the simulation of the deterministic model (Eq. 9) as $\Delta$ = 100, $\mu$= 0.5, $\psi$= 0.04,$\varepsilon$ = 0.4, $\kappa =0.8,$ $\gamma$= 0.01, $\upomega$ = 0.03, $\rho$ = 0.2,$\delta =0.3,$ $\sigma =0.04 ,$ $\varphi =1.3$, and $\beta$ = 1.4 where some of these values are related to the animosity study in^3^. Numerical simulations illustrated by the following figures show the significance of the control strategies to takle the transmission dynamics of mathematics anxiety in the students community.

**Figure 11:**
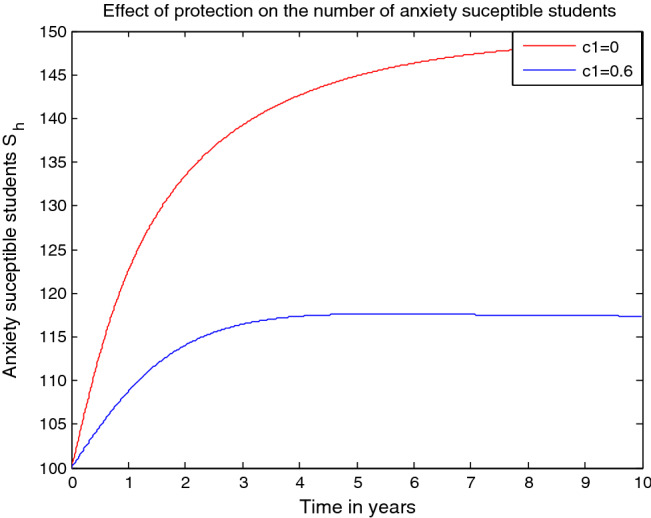
Effect of protection on the number of anxiety susceptible students ($${S}_{h})$$.

Simulation illustreated by Fig. 11 shows that when the protection strategy increases then the number of students who are susceptible to mathematics anxiety ${(S}_{h} )$ decreases while the number of students who are protected against mathematics anxiety ${(P}_{h})$ increases.

**Figure 12:**
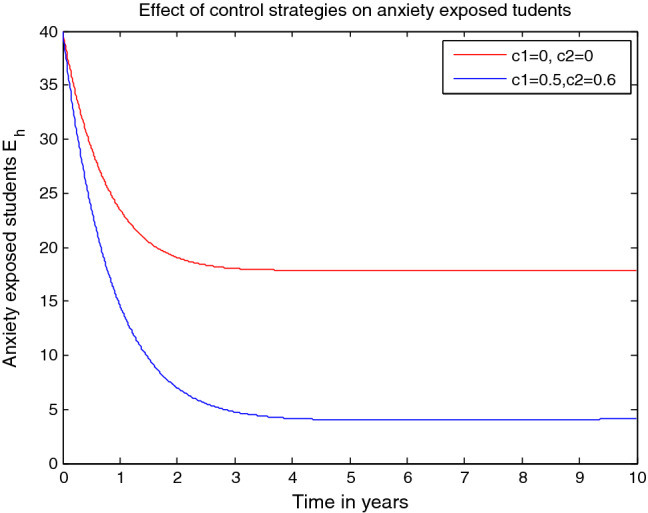
Effect of protection and treatment strategies on the number of anxiety exposed students (${E}_{h}$).

Simulation illustreated by Fig. 12 shows that when the rates of protection and treatment strategies increases then the number of anxiety exposed students ${(E}_{h})$ decreases.

**Figure 13:**
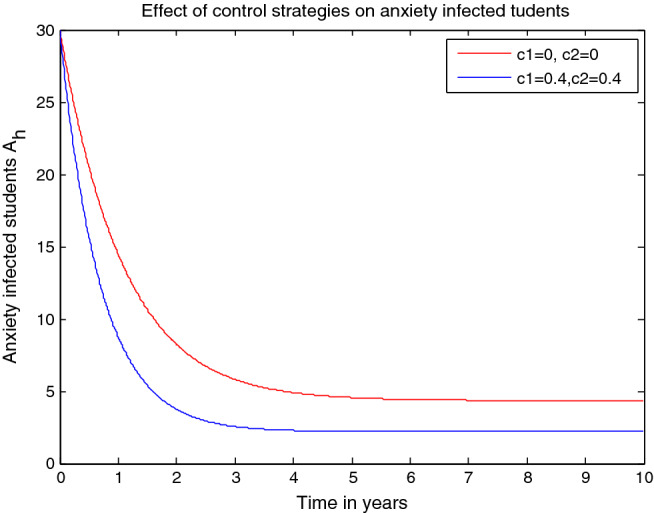
Effect of protection and treatment strategies on the number of anxiety infected students ${(A}_{h})$.

Simulation illustreated by Fig. 13 shows that when the rates of protection and treatment strategies increases then the number of anxiety infected students ${(A}_{h})$ decreases. Based on the numerical simulation results we ovserbed that applying both protection and treatment controlling strategies simultaneously has a fundamental effect on the transmission dynamics of mathematics anxiety throughout the higher institutions students community.

## Discussion and conclusion

In this study, we formulated and analyzed a deterministic mathematical model and reformulated it as a fractional order mathematical model on the higher institutions students’ anxiety towards mathematics with prevention and treatment mechanisms. In “Introduction” we have introduced the basic concepts students anxiety towards mathematics and some basic background for this study. In “Basic definitions of fractional calculus” we have recalled some basic definitions of fractional calculus which are fundamental for this resaerch study. In “Integer order model formulation” we have formulated and briefly discuss the integer order model of the transmission dynamics of higher institution students anxiety towards mathematics using a system of ordinary differential equations by dividing the total number of higher institution students population into six distinct groups. In “Model derivation in Caputo fractional order derivative approach”, we have re-formulated the integer order model given in “Integer order model formulation” into a Caputo fractional order approach and analyzed the qualitative behaviors of the model such as; non-negativity of future solutions of the model, boundedness of the dynamical system, existence of anxiety-free equilibrium point, existence of anxiety effective reproduction number using next generation matrix approach, existence of anxiety endemic equilibriums, local stability analyses of anxiety-free and anxiety endemic equilibrium points using Routh–Hurwiz and Matignon’s stability criteria for fractional oreder model. Backward bifurcation in fractional order approach is estabilished. In “Optimal control problem” an optimal control problem for the fractional order dynamical system counterpart is re-formulated and investigated. In “Numerical simulations for the deterministic model (Eq. 9)” numerical solutions for the deterministic model is carrieds out. In “Numerical simulations of the fractional order model (Eq. 12)” numerical simulation for the fractional order approach model including its counterpart optimal control problem has been performed and used to verify the qualitative (theoretical) analyses of the model. The reason for we considered a fractional order approach instead of its integer order counterpart is that the fractional order differential equation approach is a generalization of integer order differential equation. One can argue that a fractional order approach is more suitable approach than integer order approach for modelling any complex adaptive systems in different diciplines of study.

We have carried out numerical simulations for both the deterministic and fractional order approaches using MATLAB programming codes with ODE45 (the fourth order Runge–Kutta) approach to the simulation of deterministic model and with Euler forward finite difference approach for the fractional order model. From the numerical simulation results we illustrated that some parameters changes have high impacts on the anxiety effective reproduction number ${\mathcal{R}}_{eff}$ of the model and determined the result ${\mathcal{R}}_{eff}=5.41$ which shows the persistence of high anxiety in the student community, the behavior of the deterministic model solusions and effects of some influential parameters like transmission rate, protection rate and treatment rate on the model solutions, we computed the effective reproduction number of the model (Eq. 12) as ${\mathcal{R}}_{eff}^{\theta }=2.83,$ investigated the impact of memory on the number of anexiety exposed, anxiety infectious, permanently anxious and recovered form anxiety students. Also from the numerical simulation results of the optimal control problem we ovserbed that applying both the protection and treatment strategies is the most effective approach to tackle the mathematics anxiety in the students community. In general, our fractional order model numerical simulation result shows that memory has great influence on anxiety infection transmission. Even though, we suggested that the fractional order modelling approach could produce better solutions in the comparison of existing classical models, we strongly believe that this study analyses can further modified by potential researchers. However, we understand that the model analysis with fractional derivatives approach is more complicated than the classical deterministic modelling approach. To the best of my knowledge this is the first paper on higher institutions students anxiety towards mathematics in fractional order modelling approach. Eventually, since protection rate and tereatment rate have fundamental impacts to minimize the transmission dynamics of higher institution students anxiety towards mathematics we recommend for stakeholders to concentrate on maximization of both protection and treatment measures to tackle higher institutions students’ anxiety towards mathematics.

Finally, since this research study is not exhaustive any potential researcher can modified this research study by incorporating additional concepts such as the stochastic approach, age structure of students, effects of teaching aid materials, roles of parents, and fitting the model with real situations data.

## Data Availability

Data used to support the findings of this study are included in the article.
